# Discovery of Five Classes of Bacterial Defensins: Ancestral Precursors of Defensins from Eukarya?

**DOI:** 10.1021/acsomega.4c06956

**Published:** 2024-10-11

**Authors:** Marlon H. Cardoso, Lucas R. de Lima, Allan S. Pires, Mariana R. Maximiano, Peta J. Harvey, Camila G. Freitas, Rosiane A. Costa, Isabel C. M. Fensterseifer, Pietra O. Rigueiras, Ludovico Migliolo, William F. Porto, David J. Craik, Octávio L. Franco

**Affiliations:** † S-Inova Biotech, Programa de Pós-Graduação em Biotecnologia, Universidade Católica Dom Bosco, Campo Grande 79117900, Brazil; ‡ Programa de Pós-Graduação em Ciências Ambientais e Sustentabilidade Agropecuária, Universidade Católica Dom Bosco, Campo Grande 79117900, Brazil; § Centro de Análises Proteômicas e Bioquímicas, Pós-Graduação em Ciências Genômicas e Biotecnologia, Universidade Católica de Brasília, Brasília 70790160, Brazil; ∥ Institute for Molecular Bioscience, Australian Research Council Centre of Excellence for Innovations in Peptide and Protein Science, The University of Queensland, Brisbane, Queensland 4072, Australia; ⊥ Instituto Federal de Brasília, Brasília, 72620100, Brazil; # Programa de Pós-Graduação em Patologia Molecular, Faculdade de Medicina, Universidade de Brasília, Campus Darcy Ribeiro, Asa Norte, Brasília 70910900, Brazil; ∇ Porto Reports, Brasília 70790160, Brazil

## Abstract

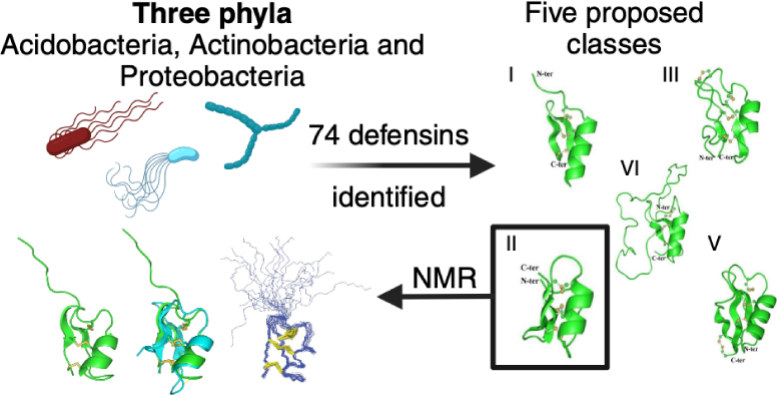

Defensins are present in many organisms and are divided into two evolutionary groups, termed *cis*- and *trans*-defensins. *Cis*-defensins have only recently been reported in bacteria, and knowledge of these defensins is limited, with no family classification. Here, we describe the identification of 74 *cis*-defensins from bacteria and propose five classes for their classification. We also report the first NMR structure determination of a *Myxoccocus xanthus* defensin, as well as its in silico expression analysis. Xanthusin-1 has a unique structure among the published defensins, which could indicate that the proposed class II peptides constitute a separate group of defensins. Xanthusin-1 gene expression was observed in casitone-based and *Streptomyces coelicolor* coculture-grown media. Our results demonstrate a wider distribution of defensins outside the Eukarya domain, shedding light on the origin and distribution of defensins. The sharing of three disulfide defensins between bacteria and eukaryotes points to a possible prokaryotic origin of the CSαβ motif. Moreover, the identification of defensins in Gram-positive and Gram-negative bacteria indicates an early origin but with many gene losses during the evolutionary process, similar to findings for eukaryotic defensins.

## Introduction

Defensins are one of the most studied groups among the disulfide-stabilized peptides and have been found widely in plants,^1^ animals,^2^ and fungi.^3^ Since the discovery of the first defensin in rabbit neutrophils, several peptides have been grouped under the name ″defensin″ and defensins are classified as α-, β-, big, CSαβ- or θ-defensins.^4,5^ Shafee et al.^6^ proposed the existence of two evolutionarily unrelated groups called *cis*- and *trans*-defensins. By this classification, *cis*-defensins would comprise CSαβ-defensins, present in invertebrates, plants, and fungi, whereas *trans*-defensins would comprise α-, β-, big, and θ-defensins, present in vertebrates and some invertebrates.^6^ Structurally, *cis*- and *trans*-defensins are differentiated both by the connectivity of the disulfide bonds and their orientation: *trans*-defensins present two opposing disulfide bonds, whereas *cis*-defensins have two parallel disulfide bonds.^6^

Considering the wide phylogenetic distribution as well as the structural similarity of *cis*-defensins, this group has been considered evolutionary-conserved. The cysteine-stabilized α-helix (CSH) motif has been identified as present in all the described *cis*-defensins.^5,7^ These peptides present a conserved tertiary structure formed by a β-sheet comprising two or three β-strands connected to an α-helix by disulfide bonds. In addition, *cis*-defensins have great sequence diversity among their members. Despite the high conservation of cysteine pairs, the general amino acid composition is quite variable with <20% identity between the sequences of the same clade.^5^ The sequence diversity reflects a wide functional variability including antimicrobial,^8^ metal chelating,^9^ proteinase and α-amylase inhibition,^10^ and cell signaling.^11^ Furthermore, the expression condition and sequence motifs appear to be highly related to peptide activity. In this way, antibacterial defensins, for example, are commonly expressed in contact with bacterial organisms.^5,12^ Some sequence motifs have been related to activities such as the γ-core in antimicrobial activity,^13^ the arthropod ion channel inhibition motif,^14^ and a highly positively charged loop in fungal cell penetration.^15^ This multiplicity of activities occurs because several members of this group evolved under different selective pressures, allowing the emergence of different activities depending on the species, tissue of expression, and/or induction.^6,12^ In addition, successive events of gene duplication may have facilitated the appearance of new mutations in genes without necessarily generating deleterious effects on their activity due to the redundancy of gene copies.^16−18^

*Cis*-defensins have been identified in several phyla. In eukaryotes, these peptides appear before the diversification of the Viridiplantae and Opisthokonta.^19^ They have been reported in both protostome clades, Ecdysozoa^7,20^ and Spiralia,^21^ as well as in plants and fungi, that have been the main sources of defensins in eukaryotes. In plants, defensin genes appear in several copies in the genomes of angiosperms and gymnosperms.^22,23^ In recent decades, databases have been explored to increase our understanding of the evolution and distribution of peptide families.^24^ Cyclotide-like peptides were identified in Poaceae plants,^24^ extending the evolutionary understanding of cyclotides – a class of plant head-to-tail-cyclized peptides – outside eudicots.^24^ In addition, heveins – a class of chitin-binding peptides – were identified in fungi species, expanding its distribution outside Viridiplantae.^25^ Recently, six families of defensins in fungi were identified and characterized, although these families have since been broadened.^19^ Despite the wide distribution of *cis*-defensins and defensin-like peptides in eukaryotes, knowledge of *cis*-defensins in prokaryotes is still expanding.^7,26^ The evolutionary origin of *cis*-defensins as a family of peptides with two disulfide bonds produced by Myxobacteraceae was also proposed.^27^ Indeed, *cis*-defensins were recently reported in bacteria.^7,26^ Nevertheless, defensin peptides outside the eukaryote domain remain underestimated, with little understanding of their distribution, structure, and classification.

Here, we propose a new identification system of bacterial defensin classes based on structural archetypes. We identified 74 *cis*-defensins in bacterial species and classified them into five distinct classes according to their structural similarities.

## Results

### Structure-Guided Identification of Bacterial Defensins

**Figure 1:**
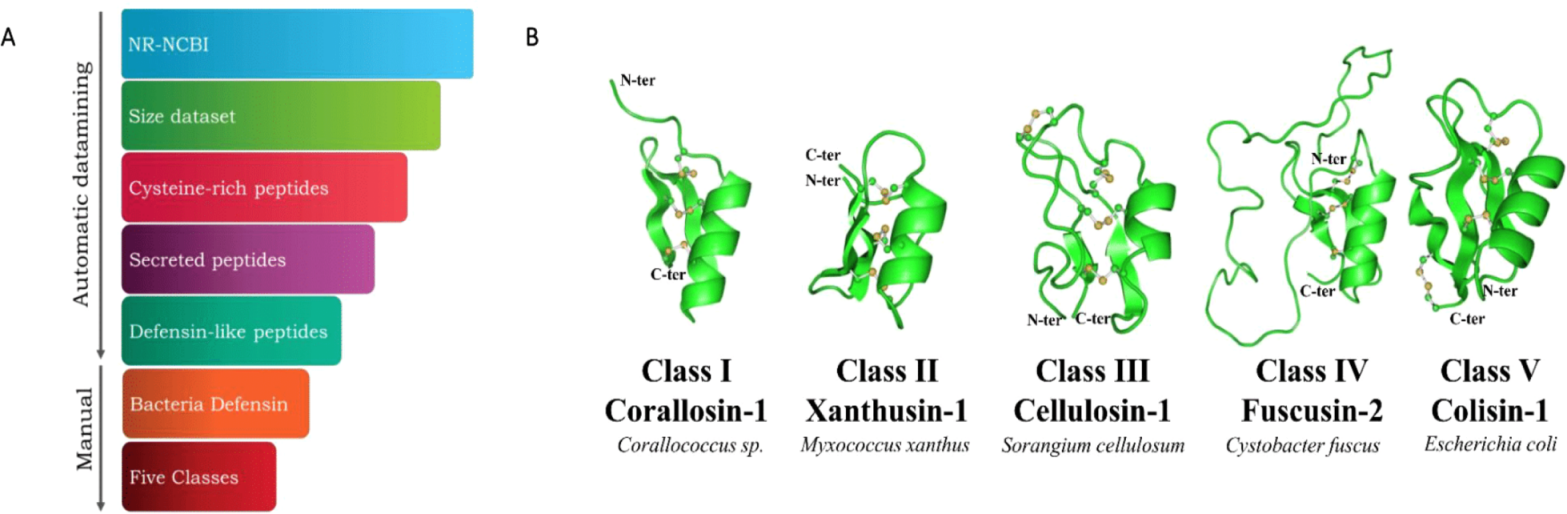
General panorama of data mining. Summary of computational workflow. (A) Initially, all sequences with more than 130 and less than 30 amino acids residues were removed, forming a size data set. This set was then evaluated by using the Zhu’s regular expression for CSαβ-defensins with modifications.^19^ The cysteine-rich database was submitted to Phobius^30^ to remove sequences with transmembrane and without signal peptide signatures, forming the secreted peptide set. The resulting set was evaluated by using HHblits secondary structure alignment to select sequences with predicted CSH folding. The predicted structure of the defensin-like classes (B). After manual verification, the bacterial defensins were grouped into five distinct classes. For each class, a peptide (see more in Figure 2) was selected for molecular modeling. A summary of the structural statistics for the theoretical models is presented in Table S1.

**Figure 2:**
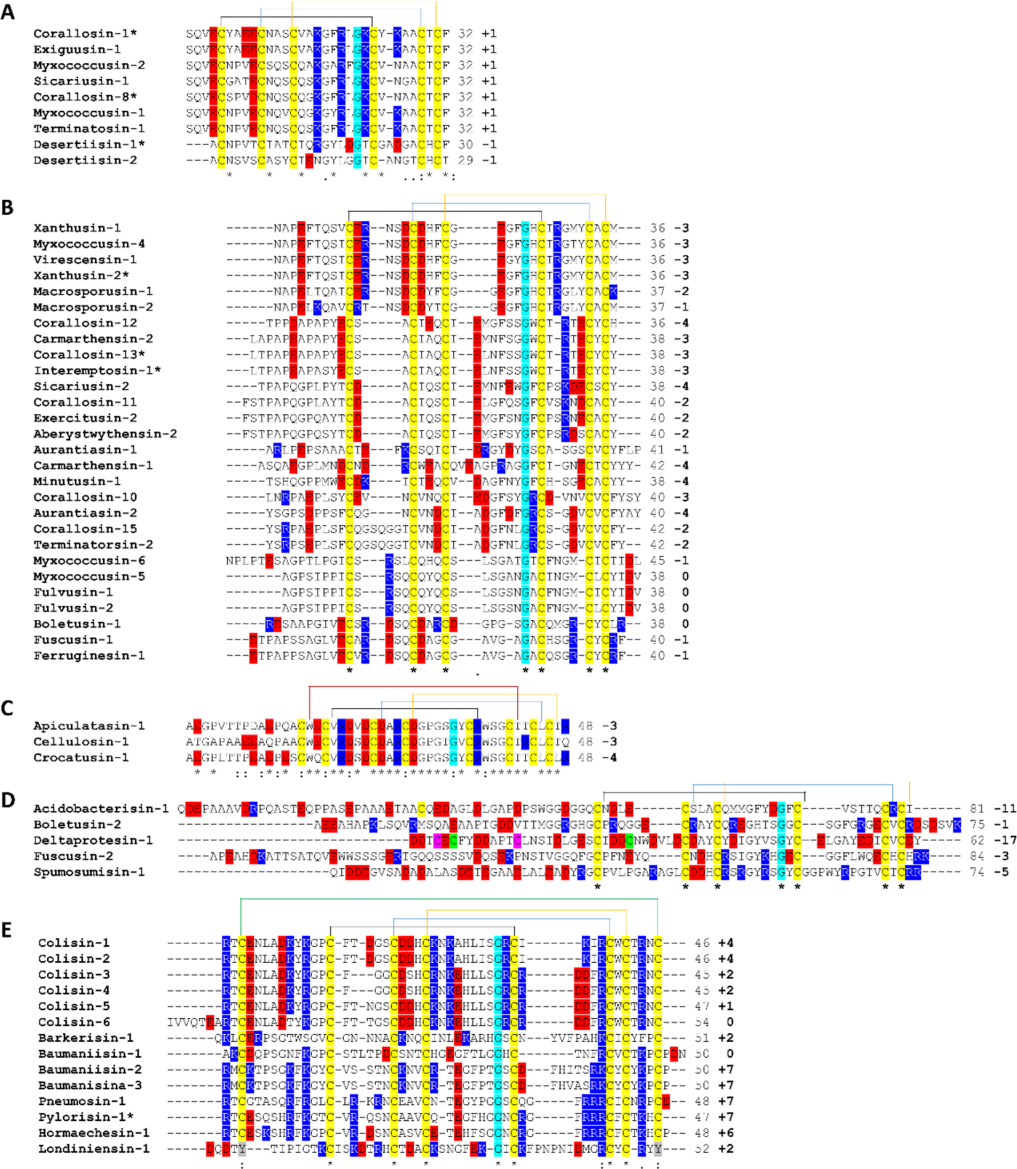
Different classes of *cis*-defensins identified in bacteria. All classes are presented by an alignment of the sequences. (A) Class I. From the 20 sequences classified as class I, 17 are from the Myxobacteraceae family, with 15 being from the *Corallococcus* genus. Moreover, the other three sequences are from *Paramesorhizobium desertii*, an α-proteobacteria. The KCXN motif is highlighted by the box in red. (B) Class II. All sequences from this class are produced by organisms of the Cystobacterineae suborder. (C) Class III. Only three sequences were classified as Class III. All sequences are from the Polyangiaceae family, two from *Chondromyces* genus, and one from *Sorangium*. (D) Class IV. The additional disulfide bonds of deltaprotesin-1 are shown in magenta and green. (E) Class V. Except for bakerisin-1, all class IV sequences are from γ-Proteobacteria. Tyrosines, highlighted in gray, replace the missing cysteine pair of londiniensin-1. Cysteines are highlighted in yellow, and conserved glycine is indicated in cyan. The disulfide bonds are presented in different colors. The negatively (aspartic and glutamic acids) and positively (lysine and arginine) charged amino acids are colored red and blue, respectively. On the right side, sequence length and liquid charges of each peptide are shown. Only Arg, Lys, Asp, and Glu residues were considered for liquid charge calculation. The NR-NCBI IDs of each sequence are presented in Tables S2–S6. *Duplicate mature sequences were omitted.

We explored the distribution of defensin-like peptides in all three life domains and mined all sequences available in the nonredundant protein database from NCBI^28^ (referred henceforth as NR-NCBI). The workflow (Figure 1A) was applied in NR-NCBI each two years between 2015 and 2021 to identify the total number of sequences. We identified 9449 defensin-like peptides up to 2021. We selected sequences identified in Eubacteria for detailed analysis. From the 194 peptides structurally aligned with defensins, 74 were predicted as defensin-like peptides. The other 94 sequences were discarded as lacking a complete CSH motif. From these remaining 74 sequences, five structural archetypes were identified and classified into five distinct classes, named I to V (Figure 1B). For classification, structural similarities such as the length of loops c, m, and n; the presence of additional disulfide bonds; the formation of additional structures to the CSH motif (such as an additional β-strand); and HHblits^29^ alignments were used. In this scheme, class I peptides present a short n loop and from one to four residues in the *N*-terminal tail. Class II, by contrast, is characterized by a 9-13 residue *N*-terminal tail. In turn, class III presents a fourth disulfide bond between cysteines 1 and 6. Class IV is classified by a long *N*-terminal tail ranging from 21 to 53 amino acids. Lastly, class V peptides have a fourth disulfide bound between cysteines 1 and 8, as well as a third β-strand, similar to plant defensins. The defensins were named by using the specific epithet as reference (Figure 2).

### Distribution of Defensin-Like Peptides in Bacterial Species

We identified 74 defensin-like peptides in three bacterial phyla. The majority belonged to Proteobacteria species with only acidobacteresin-1 (from class IV) and barkerisin-1 (from class V) belonging to Acidobacteria and Actinobacteria phyla, respectively. In addition, the five different bacterial defensin classes were distributed among Proteobacteria classes, with the δ class being the most prevalent. This taxon has 55 sequences from four of the five classes of defensins described here. Furthermore, classes II and III are exclusively δ-Proteobacteria.

Class I defensins were identified in α- and δ-Proteobacteria species with 3 and 17 sequences, respectively. This class had the highest number of identical mature peptides, with only nine unique mature sequences (Table S2). Fifteen of the sequences identified are produced by *Corallococcus* species (Figure 2A). With 30 sequences, class II bacterial defensins had the largest number of sequences among the five classes. Three pairs showed the same mature sequence but did not share the same signal peptide sequence (Table S3). All defensins identified in this class belonged to the suborder Cystobacterineae (Figure 2B). Class II defensins were identified in six genera, *Corallococcus* sp. and *Myxococcus* sp. from the family Myxococcaceae and *Cystobacter* sp., *Hyaliangium* sp., *Melittangium* sp., and *Stigmatella* sp. of the Archangiaceae family. Peptides from classes III and IV were identified only in poorly studied species, which could cause subsampling of proteomic data from these organisms. Consequently, classes III and IV defensins were found in only a few species. Class III peptides were identified only in two genera of the Polyangiaceae family (Figure 2C, Table S4). We identified five class IV defensins, with three from Archangiaceae, one from Polyangiaceae, and one from an unidentified Acidobacteria, the only peptide identified from Acidobacteria phylum (Figure 2D, Table S5). Class V was mostly identified in the Proteobacteria phylum. With the exception of barkerisin-1, all peptides were identified in human pathogenic bacteria of ε- and γ-Proteobacteria classes; barkerisin-1 was the only sequence identified in Gram-positive bacteria (Table S6). Class V peptides had the characteristic disulfide framework of plant defensins, with a fourth bond linking the first and last cysteines (Figures 1C, 2E). The only exception was longendiniesin-1, which has only three disulfide bonds (Figure 2E). Despite the absence of the fourth disulfide, this peptide has a secondary structure similar to the three-disulfide-bridged *Arabidopsis thaliana* defensin PDFL2.1 in HHblits alignments.

### Structural Characteristics of Bacterial Defensin-like Peptides

To understand the structural similarities among the defensins, representatives of each class were selected for three-dimensional structure prediction (Figure 1B) and evaluated by molecular dynamics simulations. All sequences identified presented the GXC motif between β1 and β2, configuring the presence of an γ-core (Figure 2). In addition, all classes had the characteristic three disulfide framework of *cis*-defensins. However, an additional disulfide bond was predicted for classes III and V (Figure 2) between cysteine residues 1 and 5 and between 1 and 8 for classes III and V, respectively.

The presence of an *N*-terminal tail lacking an apparent secondary structure is a characteristic of class II, III, and IV bacterial defensins (Figure 1B). These *N*-terminal tails showed a high prevalence of negatively charged and hydrophobic residues (Figure 2B–D). This region is smaller in classes II and III, varying from 8 to 13 residues in class II, to 15 residues in class III. In contrast, class IV peptides have a larger loop, ranging from 23 to 53 amino acids.

**Figure 3:**
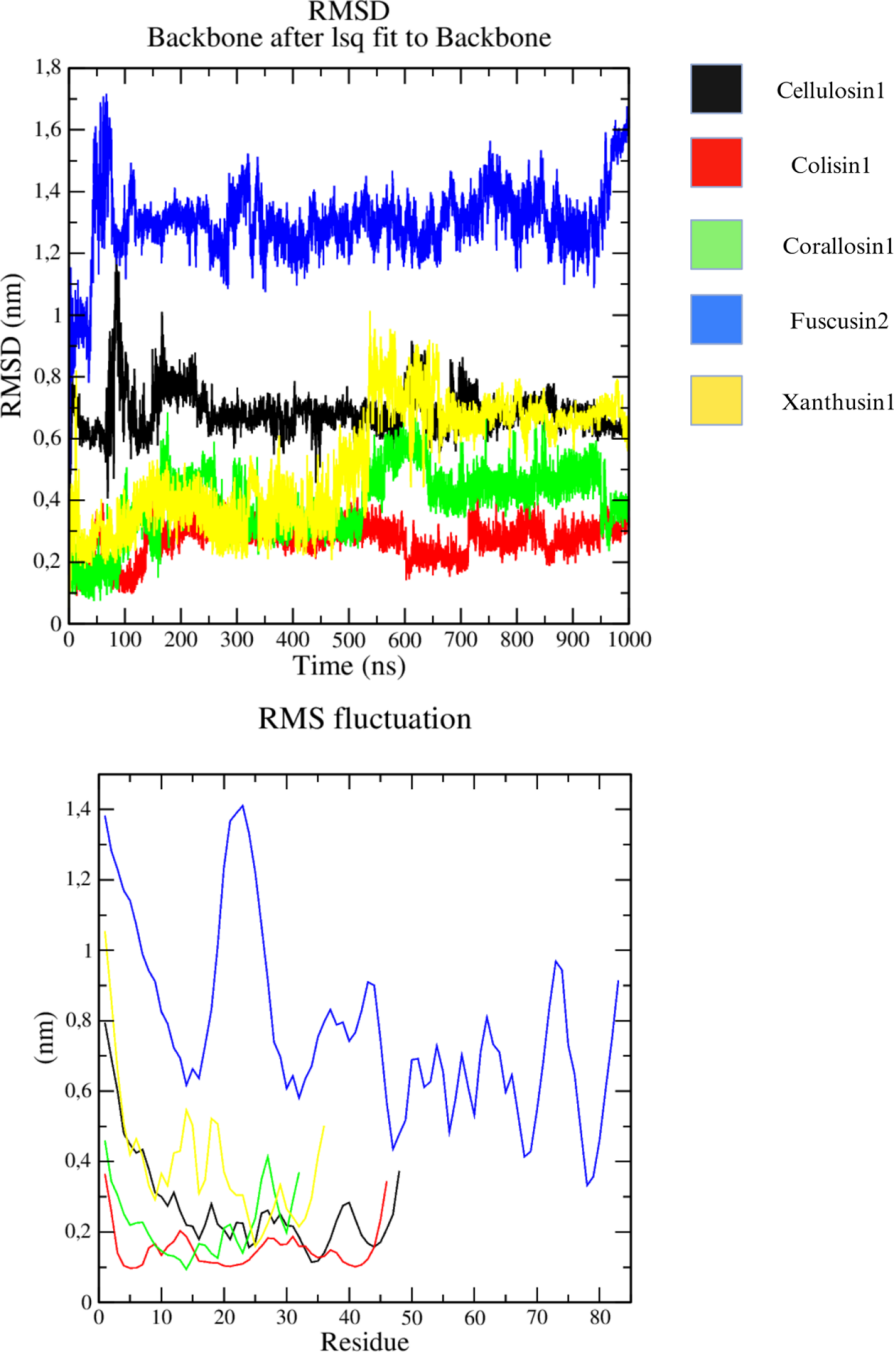
RMSD and RMSF of the bacterial defensin class prototypes. (Green) Corallosin-1, class I prototype; (yellow) xanthusin-1, class II prototype; (black) cellulosin-1, class III prototype; (blue) fuscusin-2, class IV prototype; and (red) colisin-1, class V prototype. The RMSD backbone is shown at the top and the RMSF is shown below. The RMSD represents the overall variation of the structures compared to the initial position, and the RMSF shows regions of each peptide responsible for the greatest movement.

To evaluate the structural stability of the peptides, the modeled defensins were simulated in virtual saline solution (0.2 M NaCl) for 1 μs. All peptides maintained the CSαβ-defensin scaffold during the simulations (Figure S1). Among the peptides studied here, corallosin-1 (class I) and cellulosin-1 (class III) showed the highest stability according to the RMSD data (Figure 3). The RMSF data show a large variation in the fluctuation of amino acids between the five defensins, which indicates a clear difference in the behavior of the five defensins in terms of amino acid stability. Corallosin-1 (class I) presented the greatest variation in RMSF between amino acids 25 and 30. Fuscusin-2 (class IV) presents variation in the RMSF in different areas of its structure. Altogether, these data indicate that corallosin-2 has the highest stability, whereas fuscusin-2 is the most unstable among the five defensins simulated (Figure 3).

### Xanthusin-1 is Expressed in Different Culture Conditions

To evaluate the expression pattern of a bacterial defensin, a sequence from class II was selected for further analysis. The selection prioritized the peptides from most studied strains with availability of transcriptomic data in the Sequence Read Archive (SRA). From *M. xanthus*, we identified two peptides (xanthusin-1 and −2), however, the strain specification of xanthusin-2 was not available on the database and its mature sequence differed by only one residue (Iso9 for Val9, Figure 2B). In this way, xanthusin-1 was selected.

**Figure 4:**
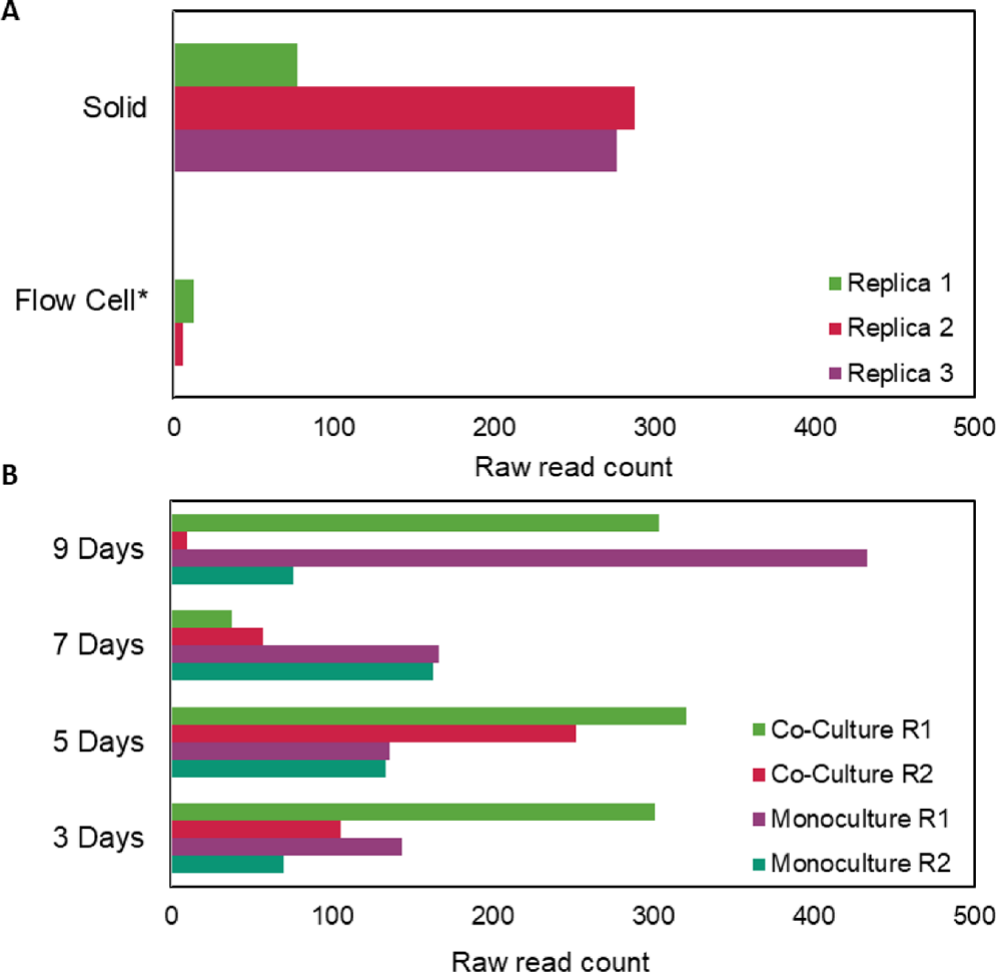
Different conditions of xanthusin-1 expression. (A) Read count of xanthusin-1 in casitone-based monoculture conditions of growth. The peptide presented reads in solid and flow cell experiments (bioproject SRP024249). (B) Read count of xanthusin-1 in coculture with *Streptomyces coelicor* and monoculture conditions in three, five, seven, and nine days (bioproject SRP180970). No difference between mono and cocultures in three and nine days was observed. *Flow cell condition was available only in duplicate.

Two SRA experiments were selected; first, growth in solid casitone-based culture media and casitone-based flow cell for four days (bioproject: PRJNA206996) and, second, coculture with the bacteria *Streptomyces coelicolor* in solid casitone-based culture media for three, five, seven, and nine days (bioproject: PRJEB25075).^31^ To validate the in silico experiments, we carried out RT-PCR experiments. The gene was expressed in all conditions tested (CTM media, CTT media, CTM 0.08%, CTM 1% with coculture with *Escherichia coli*, and LB media, at 32 °C for 7 days; Figure S2). Xanthusin-1 expression was observed to be greater in solid casitone-based culture media than in the flow cell (Figure 4A). In addition, xathusin-1 expression was also observed in coculture with *Streptomyces coelicor*. However, the presence of *Streptomyces coelicor* does not impact the peptide expression (Figure 4B). Therefore, xanthusin-1 appears to be more expressed in low nutrient conditions, such as solid growth media.

### Xanthusin-1 Adopts a Typical CSαβ Fold

**Figure 5:**
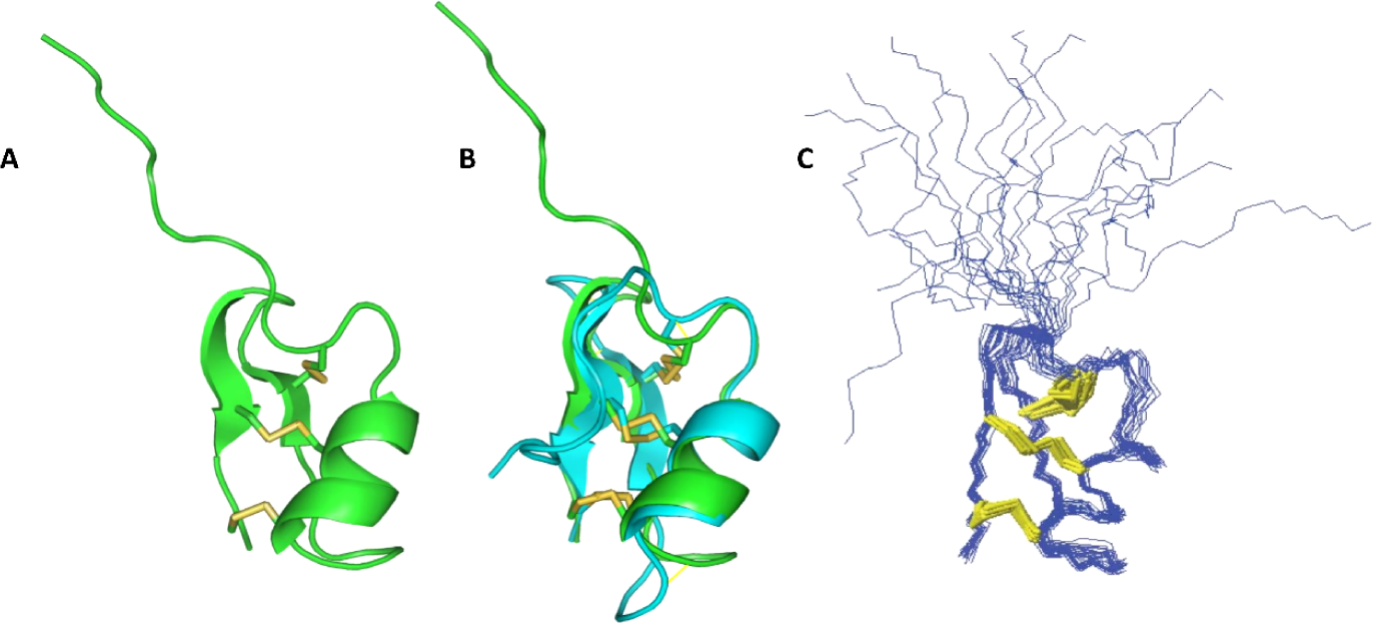
Three-dimensional structure resolved by means of nuclear magnetic resonance. (A) Cartoon representation of the xanthusin-1 structure. (B) Overlap of solved (green) and predicted (cyan) structures of xanthusin-1. Despite the differences in the *N*-terminal tail (residues 1–9) and in loop c (residues 20–25), both structures share the same core. The structures presented an RMSD of 1.47 and a 0.54 TM Score, which indicates the same structure. (C) Structural ensemble of 20 lowest energy structures, showing the backbone superimposed over residues 10–36. The disulfide bonds are shown as yellow sticks.

To analyze the structural scaffold of xanthusin-1, the synthetic peptide was evaluated by nuclear magnetic resonance (NMR). The 2D spectra were readily assigned, and 3D structure calculations performed. The 20 lowest energy structures align well, with an RMSD of 0.64 ± 0.16 Å across the backbone of residues 10–36. The stereochemical quality of the solved structural ensemble is reliable, with an overall MolProbity score of 1.3 (Table 1). The *N*-terminal tail, consisting of the first nine residues, appears flexible, with no evidence for any secondary structure. In contrast, the remainder of the peptide is well-structured and adopts a clearly defined CSαβ motif (Figure 5). Analysis of the structure by the program PROMOTIF defines an α-helix between Asn13 and Phe19 that is tethered by the three disulfide bonds to the two antiparallel β-strands formed from Gly25 to Ile28 and from Tyr32 to Cys35. The disulfide bond between the cysteine residues 1 and 4 is poorly defined due to the flexibility of the *N*-terminus, while the disulfide bond between cysteines 2 and 5 is a right-hand hook and the 3rd–4th bond is a left-hand spiral. To understand the similarities of xanthusin-1 with eukaryotic defensins, the experimental structure was evaluated by using the Dali server.^32^ However, the searches returned no hits against the PDB.

**Table 1:** NMR Structural Statistics for Xanthusin-1^a^

Experimental restraints	
total no. of distance restraints	340
intraresidue	101
sequential	129
medium range, *i* – *j* < 5	57
long-range, *i–j* ≥ 5	53
hydrogen bond restraints	14
dihedral angle restraints	
phi	24
psi	16
chi1	19

Deviations from idealized geometry	
bond lengths (Å)	0.011 ± 0.000
bond angles (deg)	1.036 ± 0.043
impropers (deg)	1.31 ± 0.11
NOE (Å)	0.010 ± 0.002
cDih (deg)	0.044 ± 0.052

Mean energies (kcal mol^–1^)	
overall	-1179 ± 28
bonds	14.5 ± 1.2
angles	36.2 ± 3.5
improper	16.4 ± 2.4
van Der Waals	-144.4 ± 4.7
NOE	0.04 ± 0.01
cDih	0.03 ±0.05
electrostatic	-1261 ± 27

Violations	
NOE violations exceeding 0.2 Å	0
dihedral violations exceeding 2.0 Å	0

Rms deviation from mean structure, Å	
backbone atoms (residues 10–36)	0.64 ± 0.16
all heavy atoms (residues 10–36)	1.43 ± 0.21

Stereochemical quality^b^	
residues in most favored Ramachandran region, %	94.6 ± 2.7
Ramachandran outliers, %	0 ± 0
unfavorable side chain rotamers, %	0 ± 0
clashscore, all atoms	3.0 ± 2.1
overall MolProbity score	1.3 ± 0.3

a All statistics are given as mean ± SD.

b According to MolProbity.

## Discussion

*Cis*-defensins are one of the oldest groups of peptides. Their wide phylogenetic distribution and varied activity of this group have led to great interest in understanding the evolutionary routes of its development. Zhu^27^ proposed the evolutionary origin of eukaryotic *cis*-defensins in a family of peptides with two disulfide bonds, called cysteine-stabilized α-helix peptides, produced by the species of the Myxobacteraceae family. In computational models, these peptides display a CSH motif similar to the *cis*-defensins, despite having only two cystine bonds to maintain the fold.^27^ However, a clear relationship between defensin-like peptides from both bacteria and eukaryotes is still missing.

Proteomic databases have previously been explored in order to increase the evolutionary understanding of the defensin family.^23,33,34^ Subsequently, several defensin groups were better described and identified such class II plant defensins^34^ and six families of fungal defensins.^33^ In this study, we used a computational approach to identify 74 bacterial defensin-like peptides structurally distributed in five different classes. Each class prototype was computationally modeled and simulated. The peptides preserved a conserved CSαβ motif during a 1 μs simulation, mainly maintained by disulfide bonds that limit core movement (Figure S1). Furthermore, substantial flexibility (from 2 to 8 Å in RMSD analyses) in loop regions was observed, which could be important for biological activity as reported in other defensins.^5,35^ In addition, the increasing number of sequences identified each year could be indicative of the subsampled groups, including bacteria, particularly on Proteobacteria. Class V defensins, for instance, were mostly identified only from the 2019 database build. Moreover, across the years, some sequences identified were removed (Table S2–S6), which could be evaluated by comparing the database builds. From the described classes, only class V represents peptides from δ-proteobacteria. During the finalization of this study, two research groups reported the presence of defensin-like peptides in the Eubacteria domain. First, Dash and coworkers,^7^ by using a similar computational approach, identified defensin-like and cysteine-stabilized α-helix peptides in many bacterial phyla including Gram-negative (e.g., Proteobacteria) and Gram-positive (e.g., Actinobacteria) groups.^7^ Second, Sugrue and coworkers^26^ isolated a defensin-like peptide named actinfensin from an *Actinomyces ruminicola* strain with antimicrobial activity. By using its sequences, the authors identified 46 other defensin-like sequences in Actinobacteria. This identification and the expression of defensin-like peptides in Actinobacteria constitute evidence of this group outside the Eukarya domain, which correlates with our data. Moreover, recently, the actinfensin (renamed AMSIN-1) structure was elucidated presenting a class I topology.^26^ The similar structures and loop lengths appear to suggest that classes I–IV are evolutionarily related (Figure 2). To understand the relation between peptides identified here and actinfensins, we applied the same classification to the sequences. Despite the great phylogenetic distance between Actinobacteria and Proteobacteria phyla, this group of actinfensins presented structural similarities to that of class I (Figure S3). These peptides were not predicted to be secreted in our analyses and were discarded during computational filtering (Table S7). Nevertheless, given the distribution of class I peptides in both Gram-negative and Gram-positive bacteria, we hypothesize that this group could represent the most ancient class of defensin peptides.

Since class II exhibited the largest number of unique mature sequences identified, we selected one defensin from this class as a structural prototype for expression and NMR analyses. The emergence of regions such as the class II *N*-terminus loop and their association with activities generated by cumulative mutations are well studied and described in *cis*-defensins.^5,12^ Thereby, we used transcriptomic data available on SRA to investigate whether the xanthusin-1 gene is expressed and, if so, under what conditions. The data indicated expression after 3 days at mono and coculture conditions, with no increase in the number of counted reads due to the presence of *Streptomyces coelicolor* (Figure 3B). However, time periods before 3 days were not available in any of the expression experiments analyzed. Moreover, conditions with higher availability of nutrients, such as flow-cell media, appear to decrease the gene expression in four day experiments (Figure 3). Due to the expression of xanthusin-1 on different conditions, the poor data on bacterial defensins expression, and the wide variety of activities described for *cis*-defenins,^8 −11^ the potential activity of xanthusin-1 could not be predicted. In general, antimicrobial defensins are highly expressed on contact with microorganisms.^5,12^ In this way, the expression pattern of xanthusin-1 was not compatible with antimicrobial defensins, such as beta-defensin, which correlates with the absence of activity in antimicrobial tests for xanthusin-1 (Table S8). This pattern was observed in an antimicrobial actinfensin produced under coculture with *S. ruminicola*.^26^

Based on our analysis of actinfensins, they appear to be class I bacterial defensins (Figure S1). From the classes proposed here, only one actinfensin^26^ (class I) and xanthusin-1 (class II) were tested for antimicrobial activity. From these peptides, only actinfensin presented antimicrobial activity.^26^ Despite the absence of antimicrobial tests, some class I peptides presented antimicrobial potential in predictions (Table S4). Moreover, four class I peptides also presented a KCXN motif (Figure 2A). This motif has been described as conserved in several arthropod α-toxin potassium channel inhibitors (α-KTx),^14^ which could indicate potential biological activity. Considering these peptides’ structures, insect defensins can be divided into two subdomains comprising a conformationally flexible amino-terminal loop (n-loop) followed by a CSαβ scaffold shared with α-KTxs. Three conserved disulfide bonds stabilize these scaffolds. In terms of function, the α-KTx in insect-derived defensins provides them not only with defense against competitors or invasive microbes by disrupting their membrane functions but also the removal of steric hindrance of peptide-channel interactions in an insect defensin structure, resulting in a Kv channel-targeted neurotoxin.^14^

Xanthusin-1 has a typical CSαβ motif (Figure 5). This peptide has the common three disulfide bond pattern found in some defensins from insects, plants, and fungi.^5^ Additionally, xanthusin-1 has a γ-core signature, as reported in the majority of defensins (Table S2).^6,13^ In contrast, xanthusin-1 had no antimicrobial activity in our tests, which reinforces the fact that the presence of a γ-core is not a guarantee of antimicrobial activity, confirming previous studies.^5,12^ The main difference between the tertiary structure of xanthusin-1 and other defensins is an *N*-terminal tail of ten residues containing no secondary structure (Figure 5). Despite the common archetype, the structure did not show hits in any Dali server searches. In addition, the Dali server search showed no similarity between xanthusin-1 and an actinfensin structure deposited in the PDB (PDB ID: 2RU0), indicating that xanthusin-1 presents a unique structure among known defensins. The dissimilarity of xanthusin-1 with known structures could indicate that class II peptides constitute a separate group of defensins (Figures S5–S7).

## Conclusions

*Cis*-defensins are an evolutionarily complex family of peptides that have been identified as being relevant in several areas of biotechnology. Until now, the identification of bacterial defensins was incomplete, and a more detailed classification was necessary. The five classes proposed here constitute an advance in the evolutionary understanding of defensins from bacteria. In addition, the structure of xanthusin-1 sheds some light on class II bacterial defensins. Moreover, the lack of similarity between xanthusin-1 and other defensin structures in Dali server searches is indicative that class II peptides compose a separate group, corroborating the classification proposed. The expression of xanthusin-1 has implications in defensin applications, as many defensins have been produced by heterologous expression using fungi and modified bacteria.^14,36^ Notwithstanding, the use of natural defensin producer strains could lower production costs. Also, since xanthusin-1 showed no antimicrobial activity, its function may resemble the multiplicity of activities of the eukaryote domain from which the three disulfide bond CSαβ motif originates. Finally, prior to the identification of defensins outside eukaryota groups, the emergence of *cis*-defensins was hypothesized to have occurred before the diversification of Viridiplantae and Opisthokonta diversification. However, given the existence of these peptides in bacteria, a more ancient origin is possible. Despite these advances in understanding, the evolutionary relationship between bacteria and eukaryotic defenses is still unclear, and it is possible to find new defensins in bacteria that may clarify their origin.

## Experimental Section

### Data Preparation and Filtering

All sequences from the nonredundant protein database (nr) were obtained from NCBI FTP site (ftp://ftp.ncbi.nlm.nih.gov/blast/db/FASTA/). Sequences were analyzed across the time period of 2015–2021. The protein sequences were filtered in four steps. First, we selected sequence lengths of 30 to 130 residues. This size range was used to represent the average sequence length with signal peptide and possible pro-regions. Second, all sequences with the cysteine pattern described by the regular expression (RegEx) CX(2,18)CX(3)CX(2,12)CX(4,17)CXC were selected (where C represents cysteine residues, X represents any of the 20 proteinogenic amino acids, and the numbers correspond to the amount of residues), with modifications.^19^ Third, we selected sequences with positive prediction by Phobius^30^ for signal peptide and negative for transmembrane regions. This step was accomplished by combining Phobius prediction. Lastly, sequences were aligned using HHblits,^29,37^ and solved structures were sourced from 70% representative Protein Data Bank (PDB – November, 2021) (http://www.rcsb.org/pdb/home/home.do). All sequences aligned with the structures of the representative database of *cis*-defensin structures. For the construction of a representative structural database, all structures from the PDB that match with the *cis*-defensin family were retrieved. The selection was performed according with Pires et al.^38^ by using the Dali server.^32^ At the end of the filtering steps, the resulting sequences were manually evaluated.

### Distribution of Bacteria *Cis*-defensins

To evaluate the distribution of *cis*-defensin in bacteria, only peptides produced by these organisms were selected for further analyses. The sequences were submitted to CD-HIT^39^ to remove redundancy. A 70% redundancy removal was applied, where for each of the groups formed, a representative sequence was selected. Representative sequences were evaluated using alignment using the Clustal Ω.^40^ The generated alignments were manually cured, prioritizing the alignment of the cysteines. The different sequences were clustered according to the length of the n, m, and c loops, as well as the disulfide bond pattern. The existence of additional bonds, secondary structures (such as the additional β-strand in β-sheet), and the HHblits alignments were also considered for classification. Using the analysis, the bacteria *cis*-defensins were distributed in five classes.

### Comparative Molecular Modeling

The comparative molecular modeling of each sequence was performed using MODELER v.9.16^41^ using the three templates from HHblits analysis. The best templates were selected according to the coverage of the sequence and the cysteine pairs. The modeling was performed by using a MODELLER automodel class. For disulfide connections not covered in templates, special patches of the automodel class were used. A total of 3000 models were generated for each peptide, and from these, one hundred models were selected according to the DOPE (Discrete Optimized Protein Structure) score. The best model (from 100) was selected by QMEAN^42^ evaluation. The best model was evaluated by PROSA II,^43^ and stereochemical quality was evaluated by PROCHECK^44^ and QMEAN.^42^ Pymol Molecular Graphics System, Version 1.6, Schrodinger, LLC, was used to visualize structures. Structures not approved in the validation steps were refined using loop refinement within MODELLER to generate 1000 new models prior to reevaluation.

### Molecular Dynamics Simulation

The molecular dynamics simulations were performed with the GROMACS 2020.4 computational package using the CHARMM27 force field.^45^ The simulations were conducted in a Verlet cutoff scheme with processing shared between CPU and GPU. Structures were immersed in water cubic boxes with 8 Å distance between the edge of the box and the structure. The simulations were performed under ionic strength conditions (0.2 M NaCl). The box was filled using the Single Point Charge water model.^46^ Additional ions were inserted into the systems to neutralize the charge. The geometry of water molecules was constrained by using the SETTLE algorithm.^47^ Atomic connections were made through the LINCS algorithm.^48^ Electrostatic corrections were made by the particle mesh Ewald algorithm, with 1.4 nm cutoff to minimize the computational time. The same cutoff radius was applied for van der Waals interactions. The steepest descent algorithm was applied to minimize system energy for 50 000 steps. After the energy minimization, the temperature (NVT ensemble) and pressure (NPT ensemble) systems were normalized to 300 K and 1 bar, respectively, each at 100 ps. The velocity-rescaling thermostat and the Parrinello–Rahman barostat were used for normalization of the temperature and pressure, respectively. After obtaining systems with energy minimized and temperature and pressure balanced, molecular dynamics (MD) simulations were carried out with a duration of 1000 ns. The complete system simulation was performed in 1 μs using the Leap-Frog algorithm as an integrator. Molecular dynamics simulations were analyzed using backbone root-mean-square deviation (RMSD) and root-mean-square fluctuation (RMSF) was analyzed using the g_rms in functions of the GROMACS package. Secondary structure conservation was assessed using DSSP version 2.0.4. Visualizations were made using a Pymol Molecular Graphics System, version 1.6 Schrödinger, LLC. The essential dynamics were performed using the g_covar and g_anaeig utilities for the GROMACS package.

### Selection of Prototype Defensin

Prototype defensin selection was performed based on the amount of data on the producing organism available in the literature. Thus, for this purpose, the existence of genomic and transcriptomic data available in public databases was evaluated. A peptide from *Myxococcus xanthus* DK1622, a model organism, was selected. The selected peptide was named xanthusin-1 according to the species of the producing organism.

### Chemical Synthesis of Xanthusin-1

Xanthusin-1 was synthesized using automated solid-phase peptide synthesis with Fmoc (9-fluoenylmethyloxycarbonyl) protection, as previously described.^49^ A fully reduced peptide was collected by simultaneous cleavage of side chain-protecting groups and peptide from the resin, followed by oxidation at room temperature in ammonium bicarbonate buffer (0.1 M) for 48 h. The peptide was acidified with trifluoroacetic acid before purification by RP-HPLC.

### NMR Analysis and Structure Calculation

Xanthusin-1 was prepared for NMR analysis by dissolving (1 mg) in 500 μL of 90% H_2_O/10% D_2_O (v/v) at pH 4. Spectra were recorded at 298 K on a Bruker Avance III HD 600 MHz spectrometer equipped with a cryoprobe, including TOCSY (with an 80 s MLEV-17 spin lock), NOESY (mixing time of 200 ms), and naturally abundant ^15^N HSQC. TOCSY spectra were also acquired at variable temperatures (283–303 K). Slowly exchanging amide protons were identified by acquiring TOCSY spectra in 100% D_2_O over 24 h, followed by ECOSY and natural abundance ^13^C HSQC. Solvent suppression was achieved using excitation sculpting. Spectra were processed using Topspin 3.6 and then analyzed using CcpNMR Analysis.^50^ Chemical shifts were referenced to internal 4,4-dimethyl-4-silapentane-1-sulfonic acid. A total of 340 NOE-derived distance restraints were used to generate preliminary structures with CYANA. Additional restraints included disulfide bonds, hydrogen bonds as indicated by slow D_2_O exchange and amide proton temperature coefficients, χ_1_ restraints from ECOSY and NOESY data, and backbone ϕ and ψ dihedral angles predicted by the program TALOS-N.^51^ Final structures were then generated within CNS^52^ using torsion angle dynamics, refinement and energy minimization in explicit solvent, and protocols as developed for the RECOORD database.^53^ A final set of structures was chosen to represent xanthusin-1 after assessment of the stereochemical quality using MolProbity.^54^ The structure of xanthusin-1 was deposited in the RCSB Protein Data Bank (PDB ID: 7UNX) and chemical shift data in the Biological Magnetic Resonance Data Bank (BMRB entry: 31010).

### Bacteria Strain and Culture Conditions

*Myxococcus xanthus* DK1622 was kindly provided by Singer’s Lab at the University of California, Davies. The strain was cultivated in solid CTTYE medium (1% casitone, 0.2% yeast extract, 10 mM Tris-HCl [pH 7.6], 1 mM KH_2_PO_4_, 8 mM MgSO_4_, 1.5% agar) and CTM medium (1% or 0.008% casitone, 10 mM MOPS [morpholinepropanesulfonic acid pH 7.0], 1 mM KH_2_PO_4_, 8 mM MgSO_4_, 1 mM CaCl_2_, 1.5% agar).^55^ The plates were incubated at 32 °C for 7 days.

### RNA Extraction and cDNA Synthesis

The bacteria biomass was harvested from the plates and transferred to centrifuge microtubes, and the total RNA was extracted using Trizol reagent (Invitrogen), according to the manufacturer’s protocol. RNA quantification was performed using the Fluorimeter method Qubit RNA Assay Kit (Invitrogen). The integrity and purity of total RNA were confirmed by electrophoresis with 1% agarose (Sigma-Aldrich, St Louis, MO, USA) and ethidium bromide (Invitrogen, Carlsbad, CA, USA). Before cDNA synthesis, RNA was treated with Turbo TM DNase (Applied Biosystems/Ambion, Foster City, CA, USA), according to the manufacturer’s instructions, to eliminate any possible contamination with genomic DNA. The cDNA was synthesized from 1 μg of total RNA using the kit Go Script Reverse Transcription System (Promega, Madison, WI, USA) according to the manufacturer’s instructions. The cDNA samples were stored at −20 °C for further analyses.

### Expression Analysis by RT-PCR

A RT-PCR experiment was performed to confirm the expression of the Xantusin-1 gene. The RT-PCR experiments were performed in a thermo cycler (Veriti 96 Well Thermal Cycler, Applied Biosystems) using the primers Xantusin-1F (GTCACGCGATGAAGAAGAAT) and XantusinR (GAAGTGGTCACAGTCCGAGT). Each reaction mixture contained 4 μL of reaction buffer 5× (Gota Buffer, Promega), 1 μM of each primer (forward and reverse), 250 μM of dNTPs, 2.5 mM of MgCl_2_ and 2.5 U of DNA Polymerase (GoTaq DNA Polymerase, Promega), and 2 μL single-stranded cDNA corresponding to each sample. The PCR program used was a step at 95 °C for 3 min to activate the Taq polymerase enzyme (hot start), 95 °C for 30 s, 60 °C for 30 s, 72 °C for 40 s repeated for 35 cycles, and 72 °C for 5 min to final extension. The amplicon products were visualized in electrophoresis (158 pb) with 1% agarose (Sigma-Aldrich, St Louis, MO, USA) with ethidium bromide (Invitrogen, Carlsbad, CA, USA). The reactions products were purified using ExoSAP-IT enzyme (Applied Biosystems), according to the manufacturer’s instructions. The PCR products were submitted to Sanger’s sequencing using the ABI Prism Bigdye Terminator Cycle Sequencing Reaction Kit (Applied Biosystems, USA) in a 3130 Genetic Analyzer (Applied Biosystems, USA) at the DNA Sequencing Facility at the Universidade Católica de Brasília (UCB).

#### In Silico Transcriptomic Analysis

In order to evaluate the expression of xanthusin-1, we used the transcriptomic data available in the Sequence Read Archive (SRA). All transcriptomics data from the *Myxococcus xanthus* strain DK1622 were retrieved and analyzed. The bioprojects of the selected transcriptomics files are shown in Table S9. The reads were mapped against the xanthusin-1 gene (ID WP_020478070) by using Bowtie-2 tool with default parameters.

### Antimicrobial Activity Assessments

The antimicrobial activity of xanthusin-1 was evaluated against strains of *Klebsiella pneumoniae* ATCC 13 883, *Staphylococcus aureus* ATCC 25 923, and *Escherichia coli* ATCC 25 922. Chloramphenicol was used as a positive control. For this purpose, the broth microdilution protocol was used, following the determinations of M7-A10 by the National Committee for Clinical Laboratory Standards in a 96-well microplate. The relationship between the optical density at 600 nm and the number of colony-forming units (CFUs) was established by a growth curve. Thus, a final volume of 100 μL was established for the activity test with 5 × 10^5^ CFU mL^–1^ of bacteria concentration. The BIOTEK Power Wave HT equipment was used for readings taken at 600 nm every 1 h for 24 h. Concentrations of 128 μg mL^–1^ to 1 μg mL^–1^ were tested by following the serial dilution protocol.

The antifungal activity tests were performed for the fungi *Candida albicans* ATCC 10 231 and *Criptococcus neoformans* H99. The test was performed according to the growth inhibition test protocol by broth microdilution according to Clinical and Laboratory Standards Institute 64, protocol M27-A3, with modifications. The bioassays to check the fungicidal activity of the peptide against microorganisms were carried out in 96-well plates, with RPMI-1640 medium buffered with MOPS and pH 7.0, with an initial density of 2.5 × 10^3^ cells. The peptide was tested in concentrations of 128 to 1 μg mL^–1^ following the serial dilution protocol. Amphotericin B (10 μg mL) and medium without fungal cells were used as the inhibition and growth controls, respectively. The test was performed in technical triplicate. The tests were conducted for 48 h in constant agitation and 37 °C. Readings were taken at 595 nm every hour. In the 24 and 48 h times, a resuspension of the material from each well was made and rereading of them was performed to avoid aggregation of cells at the bottom of the wells. The minimum inhibitory concentration was determined as the lowest concentration, with 100% inhibition of fungal growth. In addition, 3 drops of 2 μL of each tested concentration of the peptide and controls were inoculated on Sabouraud dextrose agar (37 °C for 48 h) at 24 and 48 h. This procedure was carried out to differentiate potential fungicidal activities from fungistatic ones.

## Supporting Information Available

Structure analysis, RT-PCR experiments, and multiple sequence alignment; structure statistics, peptide sequences, antimicrobial assays, and transcriptome data (PDF)
