# Supplementary material for: Discovery of Five Classes of Bacterial Defensins: Ancestral Precursors of Defensins from Eukarya?
Source: ACS Omega. 2024 Oct 11;9(45):45297–308. doi: 10.1021/acsomega.4c06956 (PMC11561630; doi:10.1021/acsomega.4c06956)
Supplement: Supplementary file 1 — ao4c06956_si_001.pdf [file ao4c06956_si_001.pdf]

**SUPPORTING INFORMATION**

# Discovery of five classes of bacterial defensins: ancestral precursors of defensins from Eukarya?

Marlon H. Cardoso[a,bI*], Lucas R. de Lima[aI], Allan S. Pires[cI], Mariana R. Maximiano[c], Peta J. Harvey[d], Camila G. Freitas[e], Rosiane A. Costa[c], Isabel C. M. Fensterseifer[c,f], Pietra O. Rigueiras[c], Ludovico Migliolo[a], William F. Porto[c,g], David J. Craik[d] and Octávio L. Franco[a,c,f]*

[a]S-Inova Biotech, Programa de Pós-Graduação em Biotecnologia, Universidade Católica Dom Bosco, Campo Grande 79117900, Brazil;

[b]Programa de Pós-Graduação em Ciências Ambientais e Sustentabilidade Agropecuária, Universidade Católica Dom Bosco, Campo Grande 79117900, Brazil;

[c]Centro de Análises Proteômicas e Bioquímicas, Pós-Graduação em Ciências Genômicas e Biotecnologia, Universidade Católica de Brasília, Brasília 70790160, Brazil;

[d]Institute for Molecular Bioscience, Australian Research Council Centre of Excellence for Innovations in Peptide and Protein Science, The University of Queensland, Brisbane, QLD 4072, Australia;

[e]Instituto Federal de Brasília, Brasília 72620100, Brazil;

[f]Programa de Pós-Graduação em Patologia Molecular, Faculdade de Medicina, Universidade de Brasília, Campus Darcy Ribeiro, Asa Norte, Brasília 70910900, Brazil;

[g]Porto Reports, Brasília 70790160, Brazil.

[I] These authors equally contributed to this work

*Corresponding authors:

Marlon H. Cardoso; Email: marlonhenrique6@gmail.com

Octávio L. Franco; Email: ocfranco@gmail.com

## Supplementary figures:

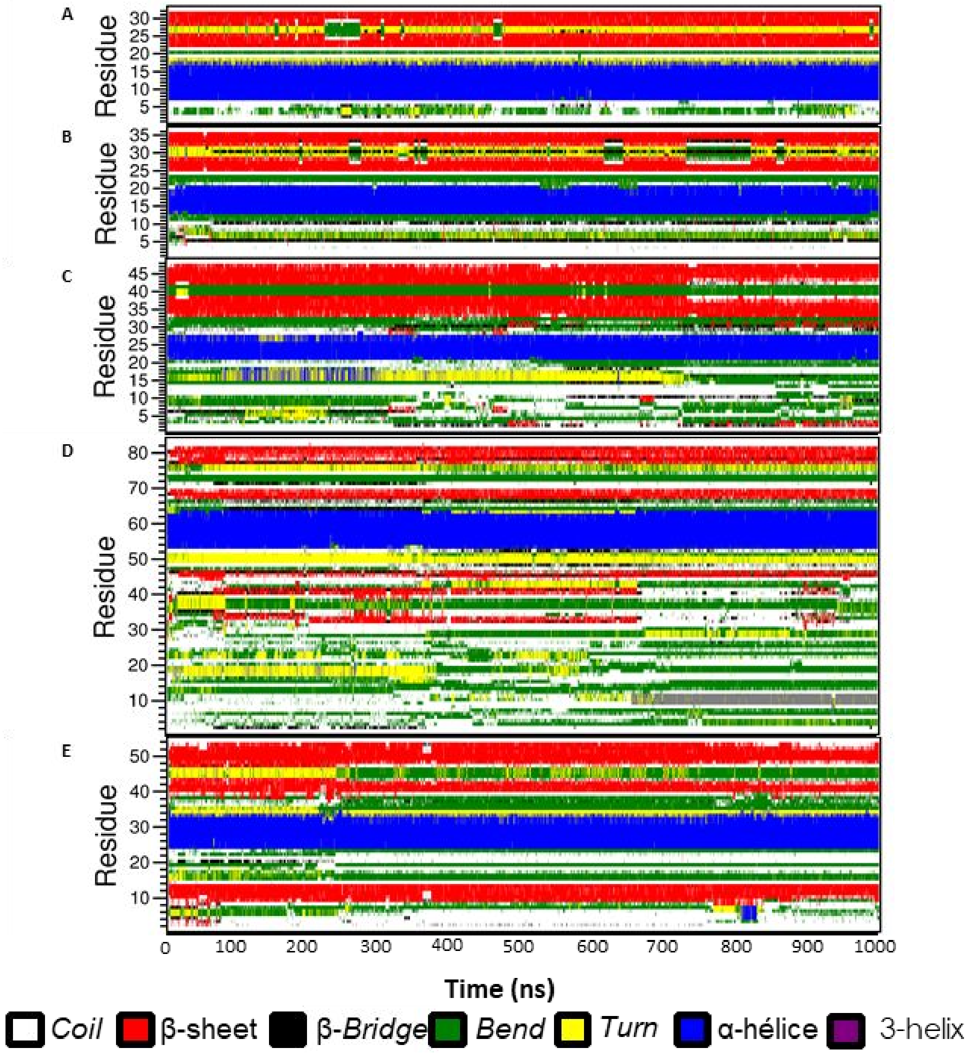


**Supplementary Figure S1. DSSP analyzes of each bacterial class prototype in 1 μs molecular simulation**. Corallosin-1 from class I (A); xanthusin-1 from class III (B); cellulosin-1 from class III (C); fuscusin-2 from class IV (D); and colisin-1 from class V (E).

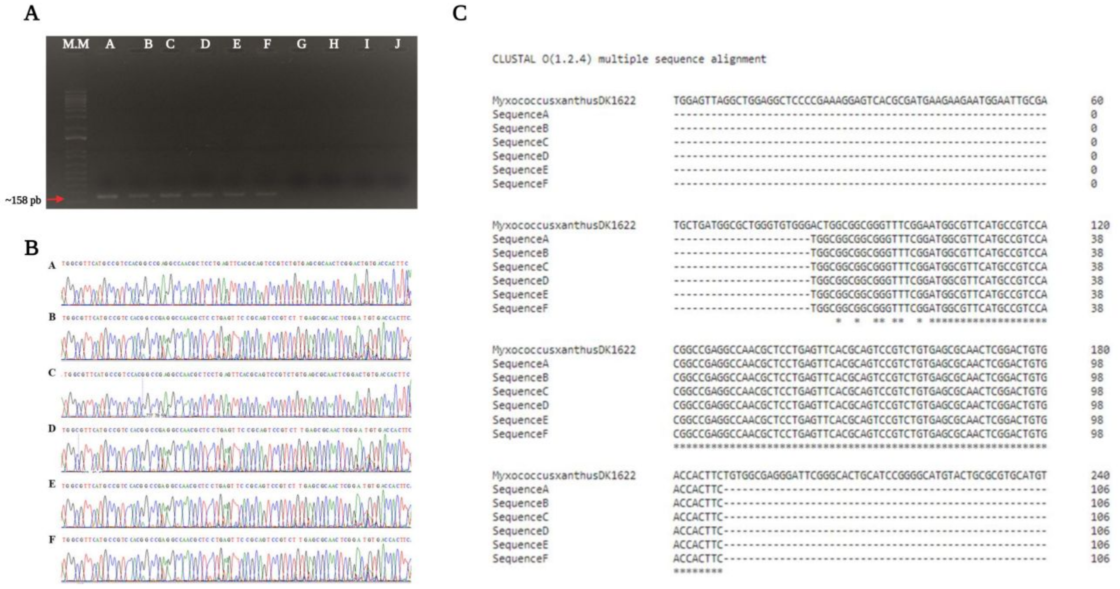


**Supplementary Figure S2. RT-PCR experiments with *Myxococcus xanthus* DK1622. A.** The expression of the xanthusin-1 gene was evaluated in five different conditions: CTM 1% media (A), CTT media (B), CTM 0.08% media (low nutrient) (C), CTM 1% media with *Escherichia coli* co-cultivation (D) and LB media (E) at 32ºC, for 7 days. The DNA of *Myxococcus xanthus* DK1622 was used as a positive control (F). The experiment had four negative control *Xanthomonas phaseoli* pv. *phaseoli* DNA (G). *Xanthomonas phaseoli* pv. *phaseoli* *c*DNA (H) *Xanthomonas campestris* pv. *campestris* (I) and Water, reaction negative control (J). Gene amplification (~158pb) was observed in all tested conditions. **B.** Sanger chromatograms (The RT-PCR products were sequenced, including positive control). **C.** Alignment of the sequencies obtained by Sanger's sequencing. All RT-PCR products were confirmed as xanthusin-1 gene.

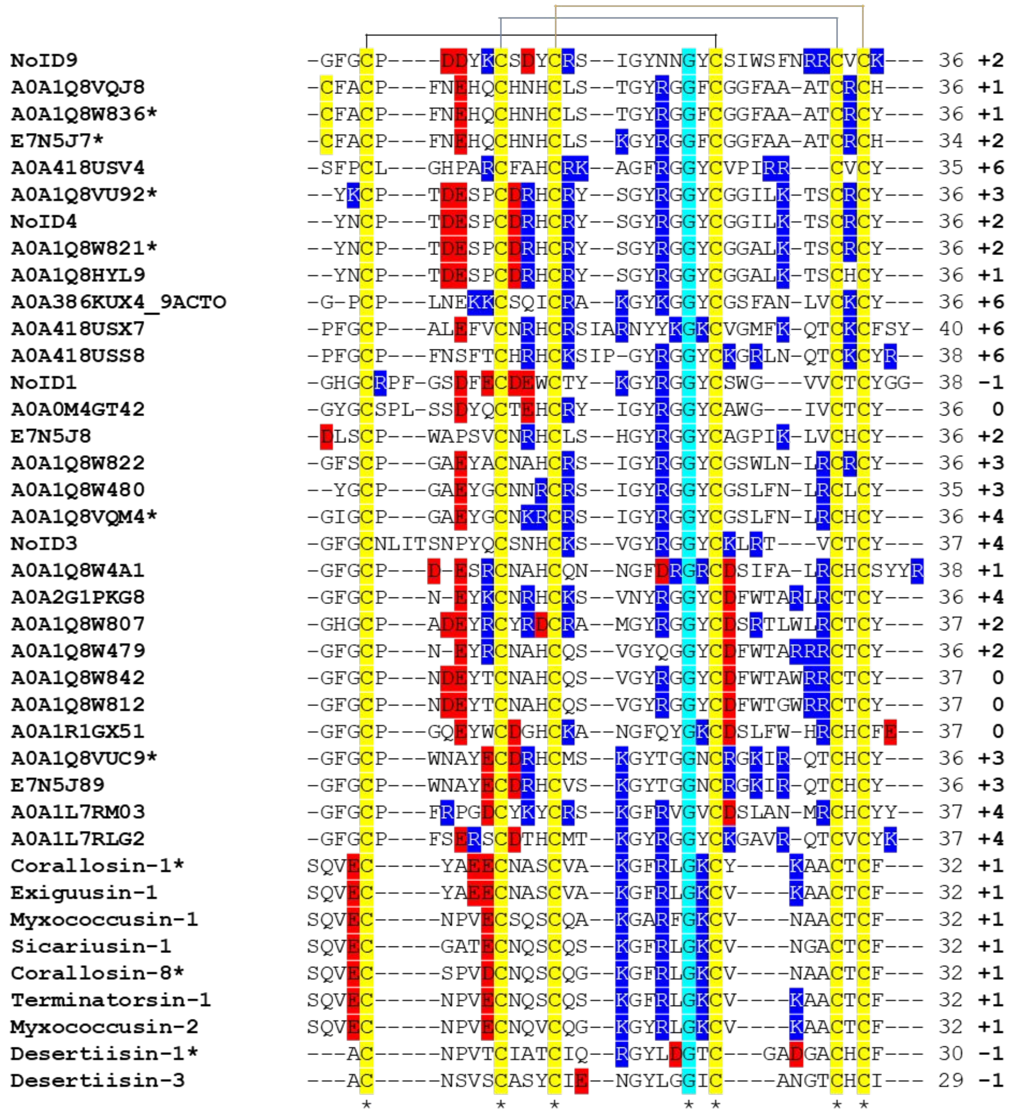


**Supplementary Figure S3. Multiple sequence alignment among actifensins and other class I defensins.** Class I peptides and actifensins are presented by an alignment of the sequences. Actifensins are defensins produced by bacteria genera *Actinomyces* by Sugrue et al. (2020). The IDs were retrieved from UniProt database and were acquired in supplementary information of actifensins description article. Sequences with no ID were named as NoID. Cysteines are highlighted in yellow, and glycines are highlighted in cyan. The disulfide bonds are presented in different colors. The negatively (aspartic and glutamic acids) and positively (lysine and arginine) charged amino acids are highlighted in red and blue, respectively. On the right side are shown the liquid charges of each peptide. Only marked residues were counted. The cleavage point of actifensins were predicted by Sugrue et al. (2020). The class I IDs are presented in Supplementary Table S2. *Duplicate mature sequences were omitted.

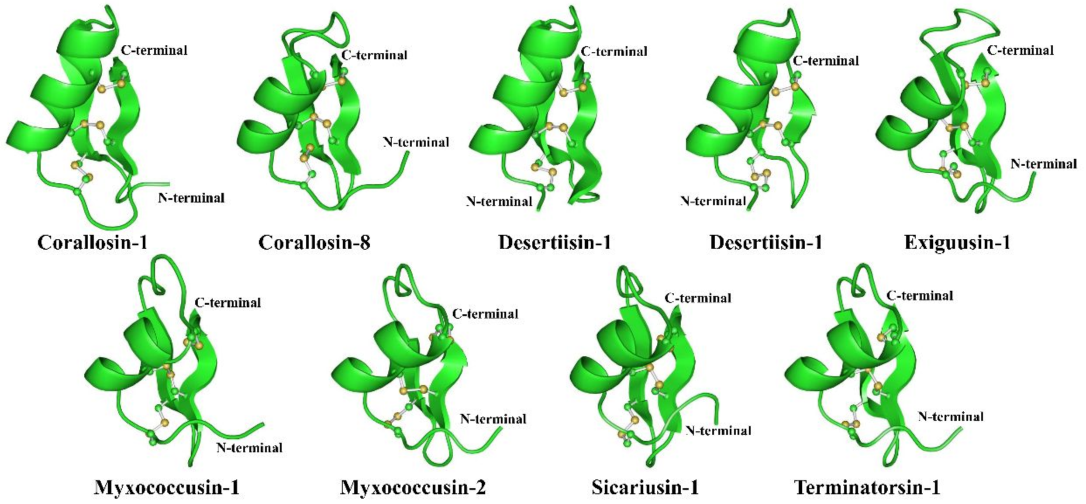


**Supplementary Figure S4. Predicted structures of class I.** Both N- and C- terminus are highlighted on each structure. The details of templates and validation steps are described on Supplementary Table S1. Structure figures were generated by using the program Pymol v1.6.

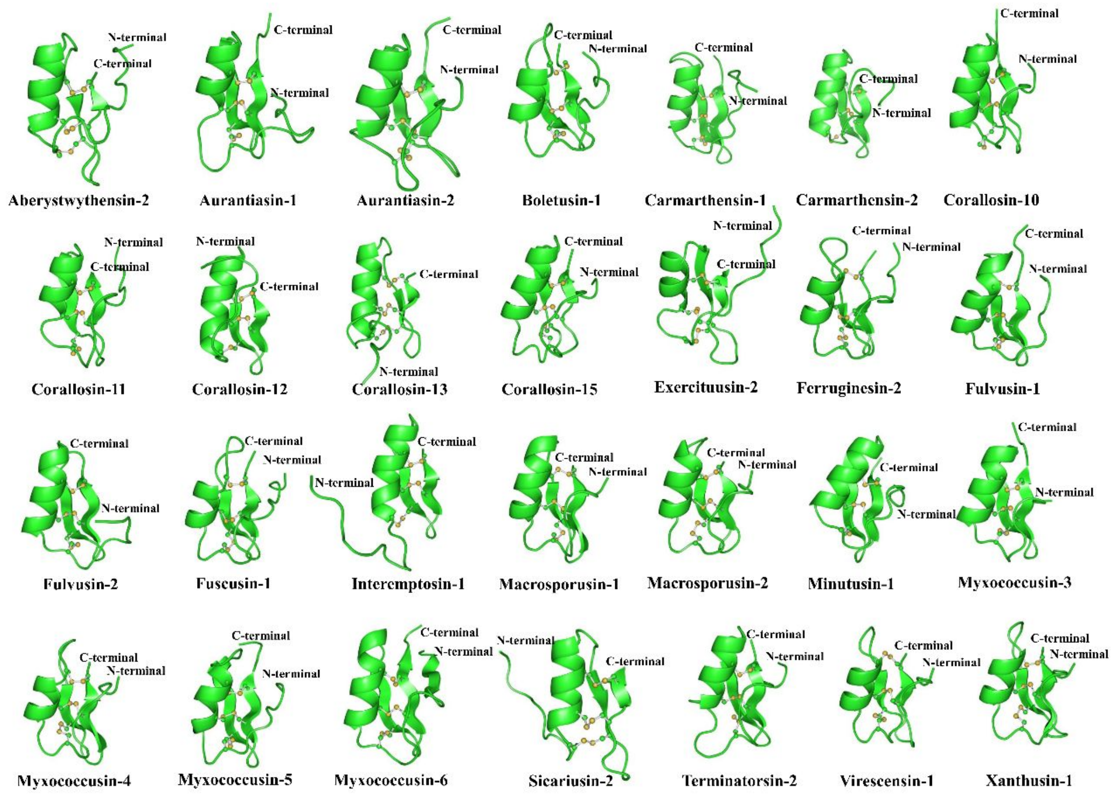


**Supplementary Figure S5. Predicted structures of class II.** Both N- and C- terminus are highlighted on each structure. The details of templates and validation steps are described on Supplementary Table S1. Structure figures were generated by using the program Pymol v1.6.

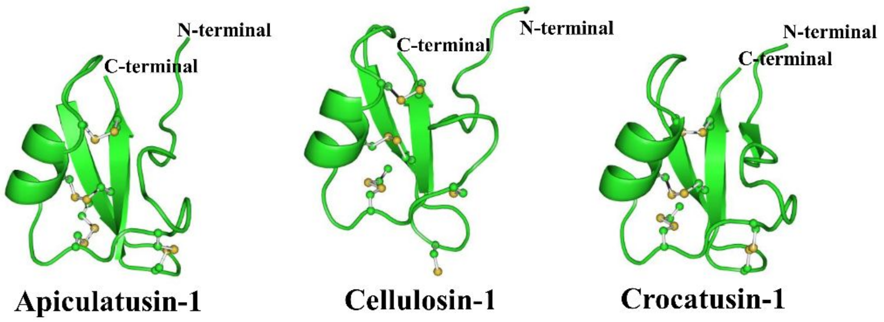


**Supplementary Figure S6. Predicted structures of class III.** Both N- and C- terminus are highlighted on each structure. The details of templates and validation steps are described on Supplementary Table S1. Structure figures were generated by using the program Pymol v1.6.

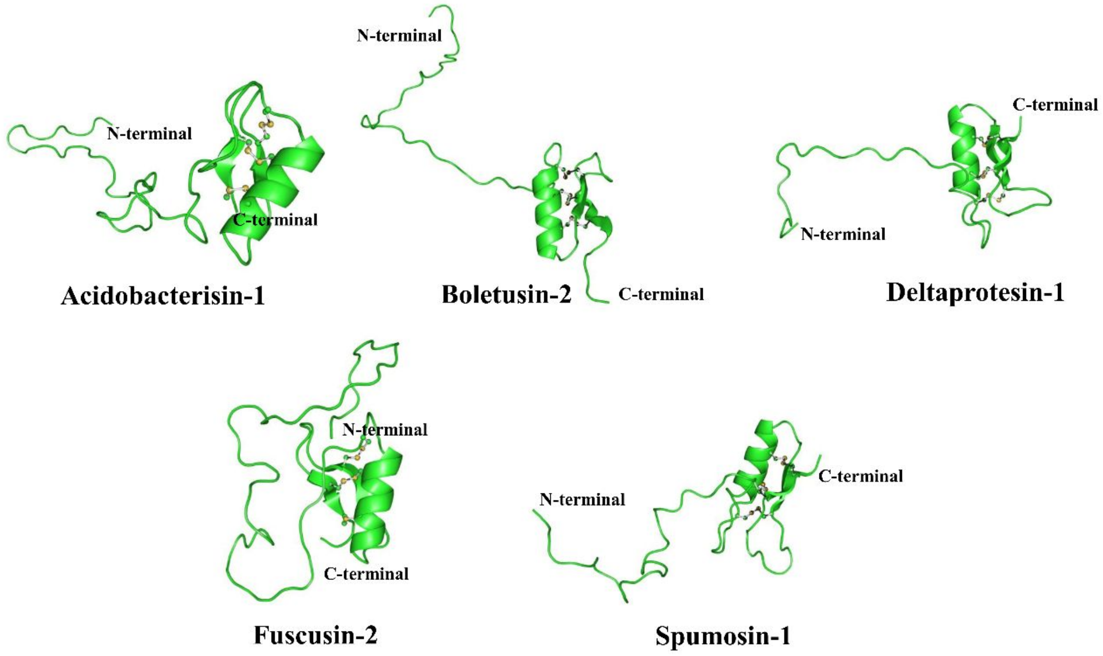


**Supplementary Figure S7. Predicted structures of class IV.** Both N- and C- terminus are highlighted on each structure. The details of templates and validation steps are described on Supplementary Table S1. Structure figures were generated by using the program Pymol v1.6.

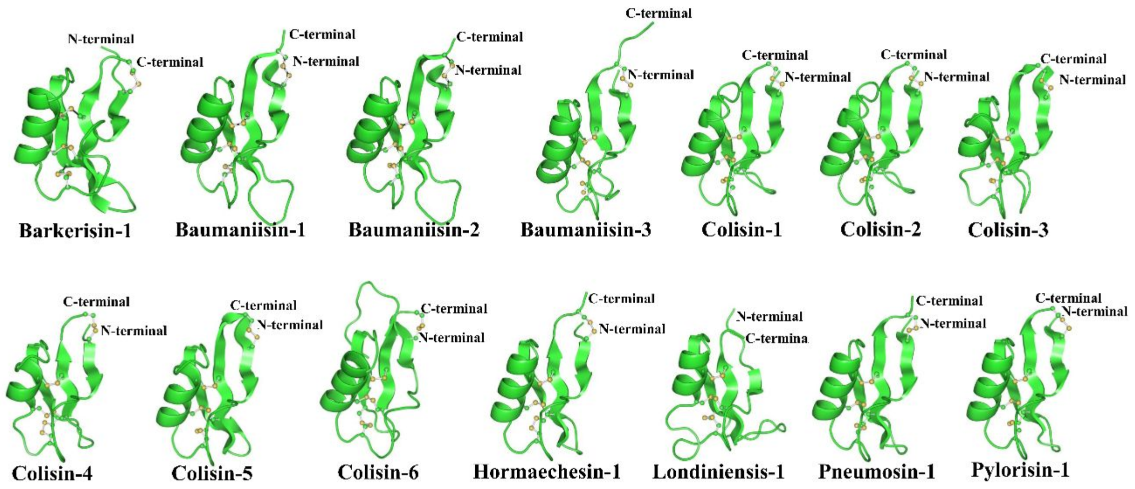


**Supplementary Figure S8. Predicted structures of class V.** Both N- and C- terminus are highlighted on each structure. The details of templates and validation steps are described on Supplementary Table S1. Structure figures were generated by using the program Pymol v1.6.

**Supplementary Tables**

**Supplementary Table S1. Summary of the structural statistics for the generated theoretical models.**

| | | Templates* | | | DOPE score | Qmean Z-score | ProSA II Z-score | Ramachandram Plot | |
|---|---|---|---|---|---|---|---|---|---|
| | | **T1** | **T2** | **T3** | | | | **Most Favoured Regions** | **Allowed Regions** |
| **CLASS I** | **Corallosin-1** | 3E8Y_A | 2KIR_A | 1PJV_A | -2276.669 | -0.042 | -7.04 | 89.3% | 7.1% |
| | **Corallosin-8** | 2KIR_A | 1M2S_A | 1LGL_A | -2166.850 | -0.001 | -7.02 | 92.3% | 7.7% |
| | **Desertiisin-1** | 2KOZ_A | 2RTY_A | 2LLD_A | -2376.279 | 0.002 | -6.55 | 95.7% | 0,0% |
| | **Desertiisin-3** | 2KOZ_A | 2RTY_A | 2LN4_A | -2316.629 | -0.002 | -7.25 | 82,6% | 17,4% |
| | **Exiguusin-1** | 2KIR_A | 2K9O_A | 1LIR_A | -1985.237 | 0.031 | -7.22 | 89,3% | 10,7% |
| | **Myxococcusin-1** | 2KIR_A | 2K9O_A | 1M2S_A | -2048.779 | 0.063 | -6.93 | 88,5% | 11,5% |
| | **Myxococcusin-3** | 2KIR_A | 2K9O_A | 1M2S_A | -1970.946 | 0.009 | -6.13 | 96,3% | 3,7% |
| | **Sicariusin-1** | 2KIR_A | 2K9O_A | 1M2S_A | -2032.567 | -0.017 | -7.77 | 96,2% | 3,8% |
| | **Terminatorsin-1** | 2KIR_A | 2K9O_A | 1M2S_A | -2040.667 | -0.165 | -6.83 | 88,9% | 11,1% |
| **CLASS II** | **Alberystwythensin-2** | 2LLD_A | 2MZ0_A | 1TI5_A | -3245.124 | -0.049 | -6.90 | 87.1% | 6.5% |
| | **Aurantiasin-1** | 1NPI_A | 1B7D_A | 1NH5_A | -2987.742 | -0.378 | -6.39 | 91.2% | 8.8% |
| | **Aurantiasin-2** | 4AB0_B | 4AAZ_B | 1GPT_A | -3098.592 | 0.116 | -5.61 | 93.4% | 3.3% |
| | **Boletusin-1** | 1JKZ_A | 3ODV_B | 2KTX_A | -2382.063 | -0.076 | -7.25 | 93.1% | 6.9% |
| | **Carmarthensin-1** | 1MTX_A | 1CMR_A | 1LIR_A | -2892.382 | 0.030 | -7.52 | 84.9% | 12.1% |
| | **Carmarthensin-2** | 2E3E_A | 2LN4_A | 2RU0_A | -2566.662 | -0.614 | -6.07 | 96.7% | 0.0% |
| | **Corallosin-10** | 1TI5_A | 4AB0_B | 4AAZ_B | -3326.724 | -0.296 | -6.19 | 88.3% | 8.8% |
| | **Corallosin-11** | 2MZ0_A | 3PSM_B | 4UJ0_B | -3019.617 | -0.486 | -6.48 | 75.0% | 18.8% |
| | **Corallosin-12** | 2RTY_A | 2KOZ_A | 2LN4_A | -2509.879 | -0.034 | -6.02 | 89.3% | 7.1% |

| | | | | | | | | | |
|---|---|---|---|---|---|---|---|---|---|
| | **Corallosin-13** | 2RTY_A | 2KOZ_A | 2LN4_A | -2705.515 | 0.051 | -5.62 | 83.9% | 12.9% |
| | **Corallosin-15** | 2LR3_A | 4AAZ_B | 4AB0_B | -3237.077 | -0.115 | -6.42 | 90.6% | 9.4% |
| | **Exercitusin-2** | 2LLD_A | 2MZ0_A | 2LN4_A | -3075.307 | -0.366 | -5.81 | 77.4% | 22,6% |
| | **Ferruginesin-1** | 1JKZ_A | 2K9O_A | 2KTX_A | -2526.134 | -0.180 | -5.88 | 96.6% | 0.0% |
| | **Fulvusin-1** | 3E8Y_X | 2KIR_A | 1LIR_A | -2923.431 | -0.000 | -5.82 | 89.7% | 10.3% |
| | **Fulvusin-2** | 1PJV_A | 2KIR_A | 3E8Y_X | -2773.188 | -0.001 | -4.82 | 89.7% | 10.3% |
| | **Fuscusin-1** | 1JKZ_A | 3ODV_B | 2KTX_A | -2526.833 | -0.247 | -6.61 | 93.3% | 6.7% |
| | **Interemptosin-1** | 2KOZ_A | 2RTY_A | 2LN4_A | -2718.195 | 0.075 | -5.56 | 93.8% | 6.2% |
| | **Macrosporusin-1** | 1P00_A | 2LR3_A | 1MYN_A | -2684.731 | -0.812 | -6.10 | 89.7% | 6.9% |
| | **Macrosporusin-2** | 1JKZ_A | 2LR3_A | 1P00_A | -2566.050 | -0.499 | -6.27 | 89.7% | 10.3% |
| | **Minutusin-1** | 1TI5_A | 4AAZ_B | 4AB0_B | -2901.150 | -0.164 | -6.25 | 86.7% | 10.0% |
| | **Myxococcusin-3** | 3PSM_B | 1NRB_A | 4AAZ_B | -2937.121 | -0.034 | -5.57 | 86.2% | 13.8% |
| | **Myxococcusin-4** | 3PSM_B | 1NRB_A | 4AAZ_B | -2597.554 | -0.181 | -7.62 | 86.2% | 13.8% |
| | **Myxococcusin-5** | 2Z3S_A | 2KTX_A | 3ODV_B | -2847.179 | -0.085 | -6.63 | 89.7% | 10.3% |
| | **Myxococcusin-6** | 1JKZ_A | 2LU9_A | 2KTC_A | -3234.625 | -0.020 | -5.09 | 100.0% | 0.0% |
| | **Sicariusin-2** | 2LLD_A | 2E3E_A | 2LN4_A | -2802.701 | -0.009 | -4.91 | 82.8% | 10.3% |
| | **Terminatorsin-2** | 4AAZ_B | 4AB0_B | 2LR3_A | -3200.947 | -0.242 | -6.10 | 93.8% | 3,1% |
| | **Virescensin-1** | 3PSM_B | 1NRB_A | 1PVZ_A | -2744.291 | -0.160 | -7.91 | 96.6% | 3.4% |
| | **Xanthusin-1** | 3PSM_B | 1N4N_A | 1I2V_A | -2747.933 | -0.051 | -8,14 | 89,7% | 10,3% |
| **CLAS S III** | **Apiculatusin-1** | 1B7D_A | 1NPI_A | 1NRB_A | -3280.139 | -0.665 | -6.12 | 78.4% | 21.6% |
| | **Cellulosin-1** | 1B7D_A | 1NPI_A | 1NRB_A | -2785,605 | 0.005 | -5,14 | 84,2% | 13,2% |
| | **Crocatusin-1** | 1B7D_A | 1NPI_A | 1NRB_A | -3299.188 | -0.549 | -6.16 | 91.9% | 5.4% |
| **CLASS IV** | **Acidobacterisin-1** | 1TI5_A | 4UJ0_B | 1GPT_A | -3776.204 | -2.192 | -4,72 | 90.6% | 9.4% |
| | **Boletusin-1** | 2B68_A | 2RU0_A | 1FJN_A | -3170.084 | -1.714 | -3.84 | 89.3% | 10.7% |
| | **Deltaprotesin-1** | 2RU0_A | 2LR5_A | 2B68_A | -3969.270 | -2.097 | -3.89 | 89.1% | 10.9% |
| | **Fuscusin-1** | 2N2Q_A | 2KSK_A | 4AAZ_B | -3706,991 | -0,042 | -4,12 | 87,0% | 13,0% |

| | | | | | | | | | |
|---|---|---|---|---|---|---|---|---|---|
| | **Spumosin-1** | 2LR5_A | 2RU0_A | 2LT8_A | -3860.479 | -1.648 | -2.84 | 79.3% | 20.7% |
| **CLASS V** | **Barkerisin-1** | 1AYJ_A | 2N2Q_A | 4AB0_B | -3760.133 | -0.975 | -4.73 | 85.7% | 14.3% |
| | **Baumaniisin-1** | 5NCE_A | 4AB0_B | 4AAZ_B | -3874.459 | -0.460 | -4.47 | 95.1% | 4.9% |
| | **Baumaniisin-2** | 5NCE_A | 4AB0_B | 4AAZ_B | -3907.103 | -0.534 | -4.39 | 90;2% | 9.8% |
| | **Baumaniisin-3** | 5NCE_A | 4AB0_B | 4AAZ_B | -3367.041 | -1.000 | -4.27 | 86.5% | 8.1% |
| | **Colisin-1** | 4AB0_B | 4AAZ_B | 1GPT_A | -3706.991 | -0,091 | -4.12 | 87.0% | 13.0% |
| | **Colisin-2** | 4AB0_B | 4AAZ_B | 1GPT_A | -3371.998 | -0.427 | -6.02 | 87.5% | 10.0% |
| | **Colisin-3** | 4AB0_B | 4AAZ_B | 1GPT_A | -3054.195 | -0.001 | -5.96 | 84.2% | 13.2% |
| | **Colisin-4** | 4AB0_B | 4AAZ_B | 1GPT_A | -3083.295 | 0.000 | -5.56 | 89.5% | 7.9% |
| | **Colisin-5** | 4AB0_B | 4AAZ_B | 1GPT_A | -3272.631 | -0.479 | -5.82 | 87.8% | 9.8% |
| | **Colisin-6** | 2N2Q_A | 2KSK_A | 4UJ0_B | -3738.076 | -0.790 | -5.69 | 97.5% | 8.3% |
| | **Hormaechesin-1** | 4AB0_B | 4AAZ_B | 1GPT_A | -3170.014 | -0.352 | -6.03 | 95.1% | 4.9% |
| | **Londiniensisin-1** | 1POO_A | 1MYN_A | 1TI5_A | -3959.785 | -0.471 | -5.79 | 88.4% | 11.6% |
| | **Pneumosin-1** | 4AB0_B | 4AAZ_B | 1GPT_A | -3415.410 | -0.008 | -5.26 | 100% | 0.0% |
| | **Pylorisin-1** | 4AB0_B | 4AAZ_B | 1GPT_A | -3265.816 | -0.252 | -7.33 | 92.5% | 7.5% |

*The PDB IDs of each template is presented with the specific chain (*e.g.*, chain A of DEF-BBB is presented as 2E3E_A).

## Supplementary Table S2. Class I peptides.

| ID | Signal Peptide | Mature Sequence | Name | NCBI Taxonomy ID | State of ID |
|---|---|---|---|---|---|
| **RKI05238.1** | *MNSMNEVCFKPKHPHRETHMMTSLKKAGRVLFAMGAGAFVSFGATLALSTPATAEA* | SQVECYAEECNASCVAKGFRLGKCYKAACTCF | Corallosin-1 | txid2316718 | Active |
| **RKI32338.1** | *MNSMDEVCFKPKHPHRETHMMTSLKKAGRVLFAMGAGAFVSFGATLALSTSATAEA* | SQVECYAEECNASCVAKGFRLGKCYKAACTCF | Corallosin-2 | txid2316714 | Active |
| **WP_120524139.1** | *MMPSLKKAGRVLFAMGAGAFVSFGATLALSTPATAEA* | SQVECYAEECNASCVAKGFRLGKCYKAACTCF | Exercitusin-1 | txid2316736 | Active |
| **WP_120546219.1** | *MMQTLKKASRVLFAMGAGAFVSFGATLALSTPATAEA* | SQVECYAEECNASCVAKGFRLGKCYKAACTCF | Corallosin-3 | txid2316720, txid2316721 | Active |
| **WP_120556869.1** | *MMTPLKKAGRVLFAMGAGAFVSFGATLALSTPTTAEA* | SQVECYAEECNASCVAKGFRLGKCYKAACTCF | Aberystwythensin -1 | txid2316722 | Active |
| **WP_120564825.1** | *MLTSLKKAGRVLFAMGAGAFVSFGATLALSTPATAEA* | SQVECYAEECNASCVAKGFRLGKCYKAACTCF | Corallosin-4 | txid184914, txid2316735, txid2316730, txid2316719 | Active |
| **WP_120586237.1** | *MMTSLKKAGRVLFAMGAGAFVSFGATLALSTPATAQA* | SQVECYAEECNASCVAKGFRLGKCYKAACTCF | Corallosin-5 | txid2316734 | Active |
| **WP_014394946.1** | *MMTSLKKAGRVLFAMGAGAFVSFGATLALSTPATAEA* | SQVECYAEECNASCVAKGFRLGKCYKAACTCF | Corallosin-6 | txid1144275, txid83462, txid2316718 | Active |
| **WP_128795726.1** | *MLTSLKKASRVLFAMGAGAFVSFGATLALSTPATAEA* | SQVECYAEECNASCVAKGFRLGKCYKAACTCF | Coralloidesin-1 | txid184914 | Active |
| **WP_147454804.1** | *MMTSLKKAGRVLFAMGAGAFVSFGATLALSTSATAEA* | SQVECYAEECNASCVAKGFRLGKCYKAACTCF | Corallosin-7 | txid2316714 | Removed |
| **WP_120624627.1** | *MRLSVKKAGRVVLAMLTGASMSFGLTLAFSTPAQA* | SQVECGATECNQSCQSKGFRLGKCVNGACTCF | Sicariusin-1 | txid2316726 | Active |
| **RYZ43773.1** | *MHLSLKKSGRVLLAMATGAALSFGATMTFSTPAEA* | SQVECNPVECNQVCQGKGYRLGKCVKAACTCF | Myxococcusin-1 | txid2484252 | Active |
| **WP_139920944.1** | *MMTSLKKAGRVLFAMGAGAFVSFGATLALSTPTTAEA* | SQVECYAEECNASCVAKGFRLGKCVKAACTCF | Exiguusin-1 | txid83462 | Active |
| **WP_147444159.1** | *MTLSVNKAGRALLAMVTGAALSFGVTMAFSTPAQA* | SQVECSPVDCNQSCQGKGFRLGKCVNAACTCF | Corallosin-8 | txid2316725, txid2316724 | Active |
| **WP_147446426.1** | *MTLSVKKAGRALLAMVTGAALSFGVTMAFSTPAQA* | SQVECSPVDCNQSCQGKGFRLGKCVNAACTCF | Corallosin-9 | txid2316729 | Active |
| **WP_147448663.1** | *MTLSVKKAGRALFAMVTGAALSFGATMAFSTPAEA* | SQVECNPVECNQSCQSKGFRLGKCVKAACTCF | Terminatorsin-1 | txid2316733 | Active |
| **RYZ46542.1** | *MNLSVKKAGRVLLAMLTGASMSFGLTLAFSTPAQA* | SQVECNPVECSQSCQAKGARFGKCVNAACTCF | Myxococcusin-2 | txid2484252 | Active |

| | | | | | |
|---|---|---|---|---|---|
| **KXF76780.1** | *MGRRFNIWRETMLAMTTLPKLADAFRATSLRRKLTIAAFALLMGAGPVAA* | ACNPVTCIATCIQRGYLDGTCGADGACHCF | Desertiisin-1 | txid1494590 | Active |
| **WP_153020249.1** | *MLAMTTLPKLADAFRATSLRRKLTIAAFALLMGAGPVAA* | ACNPVTCIATCIQRGYLDGTCGADGACHCF | Desertiisin-2 | txid1494590 | Active |
| **WP_068882350.1** | *MLAMTTLPKLADAFRATSLRRKLTIAAFALLIGAGPVAA* | ACNSVSCASYCIENGYLGGICANGTCHCI | Desertiisin-3 | txid1494590 | Active |

## Supplementary Table S3. Class II peptides.

| ID | Signal Peptide | Mature Sequence | Name | NCBI Taxonomy ID | State of ID |
|---|---|---|---|---|---|
| **WP_147450006.1** | *MIFSAKKAGQVLLAVAAGFVVSFGGTQAFS* | ASQAEGPLMNDCNDRCWEACQVEAGPRAGGFCIGNECTCYYY | Carmarthensin-1 | txid2316728 | Active |
| **SEM57968.1** | *MRQIIVGAALALGLGVGFTAGIPSAEA* | ARLPEPSAAACTEFKCSQICTDRGYDYGSCASGSCVCYFLP | Aurantiasin-1 | txid41 | Active |
| **WP_002629432.1** | *MTKQLKGLWAVAALVVGVGTGFTAGAWA* | ETPAPSSAGLVECARDSQCDAGCGAVGAGACHSGRCYCRF | Fuscusin-1 | txid1242864 | Active |
| **WP_071902991.1** | *MTKQLKSLWAVAALVVGVGMGFTTGART* | ETPAPPSAGLVECVRDSQCDAGCGAVGAGACQSGRCYCRF | Ferruginesin-1 | txid83449 | Active |
| **WP_120203099.1** | *MKKLGQLLFAMTLGAVVSFGGTQA* | LNRPAEPLSYCEVNCVNQCIEDGFSYGRCDVNVCVCFYSY | Corallosin-10 | txid2249217 | Active |
| **WP_120527547.1** | *MIRFAKNVGQTLLAVVAATAVSFTATQA* | FSTPAPQGPQAYTCDACIQSCIEMGFSNGFCPSRNECACY | Exercitusin-2 | txid2316736 | Active |
| **WP_120608902.1** | *MIHLVKEAGKTLLALVAASALSFTATQA* | FSTPAPQGPLAYTCDACIQSCIELGFQSGFCVSKNDCACY | Corallosin-11 | txid2316732 | Active |
| **WP_120627901.1** | *MIRLVRDAGQALLAVVAATALSFSATQAFS* | TPAPQGPLPYTCDACIQSCIEMNFEWGFCPSKDECSCY | Sicariusin-2 | txid2316726 | Active |
| **WP_147445527.1** | *MIRFVKNAGQTLLAVVAATAVSFSATQA* | FSTPAPQGPQSYTCDACIQSCIEMGFSYGFCPSRDSCACY | Aberystwythensin -2 | txid2316722 | Active |
| **WP_120596915.1** | *MIDFVKKAGKTLLAVATGAVLSFSATQALA* | TPPDAPAPYECSACIEQCIEMGFSSGWCTRTECYCH | Corallosin-12 | txid2316718, txid83462 | Active |
| **WP_147442598.1** | *MRGFVKKTGQTLLAVVAGTALSFSATQA* | LTPAPEAPAPYECSACIAQCIELNFSSGWCTRTECYCY | Corallosin-13 | txid2316721 | Active |
| **RKH54233.1** | *MKKTGQTLLAVVAGTALSFSATQA* | LTPAPEAPAPYECSACIAQCIELNFSSGWCTRTECYCY | Corallosin-14 | txid2316723 | Active |
| **WP_147449973.1** | *MIDFVKKAGQTLLAVVAGSALSFSATQA* | LAPAPEAPAPYECSACIAQCIEMNFSGGWCTRTECYCY | Carmarthensin-2 | txid2316728 | Active |
| **WP_147468244.1** | *MRGFVKKTGQTLLAVVAGTALSFSATQA* | LTPAPEAPASYECSACIAQCIELNFSSGWCTRTECYCY | Interemptosin-1 | txid2316720 | Active |
| **RKH72256.1** | *MKKTGQTLLAVVAGTALSFSATQA* | LTPAPEAPASYECSACIAQCIELNFSSGWCTRTECYCY | Interemptosin-2 | txid2316720 | Active |
| **WP_120531934.1** | *MNSSVKKAGKLMLAVAVGAAVSFGSTVAA* | YSRPAEPLSFCQGSQGGTCVNDCIADGFNLGRCSGDVCVCFY | Corallosin-15 | txid2316729, txid2316725, txid2316724 | Active |
| **WP_120539224.1** | *MNSSVKKAGKLLLAVAVGAAVSFGSTVAA* | YSRPSEPLSFCQGSQGGTCVNDCIADGFNLGRCSGDVCVCFY | Terminatorsin-2 | txid2316733 | Active |
| **WP_044192120.1** | *MIRIWKKSAQALLAASVGFAVSLGATQALA* | TSHQGPPMWECDKICTEQCVDAGFNYGFCHSGECACYY | Minutusin-1 | txid394096 | Removed |
| **WP_095979179.1** | *MTKHAKGLWAVGALVLGLGMGFTAGTHTEA* | RESAAPGIVECSRDSQCDARCDGPGSGACQMGRCYCLR | Boletusin-1 | txid1294270 | Active |
| **WP_013937234.1** | *MKKNGMAMLAALGVGLAAGFGMAFMPSTAEA* | NAPELTQAICERNSDCDYFCGGEGFGHCIRGLYCACK | Macrosporusin-1 | txid35 | Active |

| | | | | | |
|---|---|---|---|---|---|
| **WP_020478070.1** | *MKKNGIAMLMALGVGLAAGFGMAFMPSTAEA* | NAPEFTQSVCERNSDCDHFCGEGFGHCIRGMYCACM | Xanthusin-1 | txid246197,<br>txid1198133,<br>txid1198538,<br>txid2562800 | Active |
| **WP_090493697.1** | *MKKNGIAMLMALGVGLAAGFGMAFMPSTAEA* | NAPEFTQSICERNSDCDHFCGEGYGHCIRGMYCACM | Virescensin-1 | txid83456 | Active |
| **WP_095961140.1** | *MTKNGIAMLAALGVGLAAGFGMAFMPSTAEA* | NAPELKQAVCRTNSDCDYTCGGEGFGHCIRGLYCACM | Macrosporusin-2 | txid1189310 | Active |
| **WP_140795974.1** | *MKKNGIAMLMALGVGLAAGFGMAFMPSTAEA* | NAPEFTQSICERNSDCDHFCGEGFGHCIRGMYCACM | Myxococcusin-3 | txid34,<br>txid2651864,<br>txid2651865,<br>txid2562799,<br>txid2562798,<br>txid2562797,<br>txid2651866 | Active |
| **WP_140869366.1** | *MLMALGVGLAAGFGMAFMPSTAEA* | NAPEFTQSICERNSDCDHFCGEGFGHCIRGMYCACM | Xanthusin-2 | txid34 | Active |
| **WP_141589640.1** | *MKKNGIAMLMALGVGLAAGFGMAFMPSTAEA* | NAPEFTQSICERNSDCDHFCGEGFGHCIRGTYCACM | Myxococcusin-4 | txid2562793,<br>txid2562792 | Active |
| **WP_046710827.1** | *MYRIMMSTALMVGLGGGFTLGTFPLAAEA* | AGPSIPPICSRSQCQYQCSLSGSNGACFNGMCICYIDV | Fulvusin-1 | txid33 | Removed |
| **WP_141331950.1** | *MYRIMMSTALMVGLGGGFTLGAFPLAAEA* | AGPSIPPICSRSQCQYQCSLSGANGACINGMCLCYIDV | Myxoccusin-5 | txid2562794 | Active |
| **WP_141648459.1** | *MRRIMMSAALMVGLGGGFSVGA* | NPLPTESAGPTLPGICSRSLCQHQCSLSGATGTCFNGMCICTIDL | Myxoccusin-6 | txid2741738,<br>txid2741739,<br>txid2741740,<br>txid2741516,<br>txid2590453 | Active |
| **WP_143096924.1** | *MYRIMMSTALMVGLGGGFTLGAFPLAAEA* | AGPSIPPICSRSQCQYQCSLSGSNGACFNGMCLCYIDV | Fulvusin-2 | txid33 | Active |
| **WP_013374863.1** | *MNSSVKKAGKLLLAVAVGAAVSFATTVAA* | YSGPSDPPSFCQGNCVNDCIADGFDFGRCSGDVCVCFYAY | Aurantiasin-2 | txid41 | Active |

## Supplementary Table S4. Class III peptides.

| ID | Signal Peptide | Mature Sequence | Name | NCBI Taxonomy ID | State of ID |
|---|---|---|---|---|---|
| **KYF83680.1** | *MMRMVRRALASVILLSGVGLWVCAAGAPAAA* | ATGAPAAEEAQPAACWECVKDSDCDARCDGPGTGVCRWSGCTRCLCTQ | Cellulosin-1 | txid56 | Active |
| **WP_044238153.1** | *MKKKIQRALVSTMLLAGIGFWVGAAGSPAVA* | AEGPVTTPDAEPQACWECVRDVDCDAKCDGPGSGYCKWSGCTTCLCTR | Apiculatusin-1 | txid51 | Active |
| **WP_050435295.1** | *MKKKTTHRVLASMLLMAGMGLWASTTGTPAVA* | AEGPLTTPEAEPESCWQCVKDSDCDARCDGPGSGYCRWSGCTTCLCLR | Crocatusin-1 | txid52 | Active |

## Supplementary Table S5. Class IV peptides.

| ID | Signal Peptide | Mature Sequence | Name |
|---|---|---|---|
| **WP_095987040.1** | *MMTQKWMNGALIAVAVVLGAWSTDGIA* | APEAHDKATTSATQVEWWSSSGERTGQQSSSSVDQSEKPNSTVGGQFGCPFNEYQCNDHCRSIGYKHGECGGFLWQECHCHRK | Fuscusin-2 |
| **RMH19113.1** | *MLRTMLLRLSLLAVLVMVLAVAATAA* | QDEPAAAVDRPQASTEQPPASEPAAAETAACQEDAGLDLGAPDPSWGGDGGQCNDLECSLACQMMGFYDGFCVSTTQCRCI | Acidobacteris 1 |
| **WP_095978346.1** | *MKKTQWRALALAPMVFILSVCGG* | AEEAHAPKLSQVRMSQAEAAPTGDDVTTMGGRGHGCPRQGGECRAYCQREGHTSGGCSGFGRGECVCRDSDSVK | Boletusin-2 |
| **WP_153823935.1** | *MLKKMLCVLMLAVGAGACAVDA* | QIDDEGVSADADALASDETEGAAELALEADYRGCPVLPGARAGLCDDHCRSRGYRSGYCGGPWYRPGTVCTCRR | Spumosin-1 |
| **PKN23827.1** | *MKKLFAAALCMGFFATACVVT* | DDTCECFYDDAPTCLNSIDLGESCIDDCNWDVLDCDAYCYDIGYVSGYCELGAYEDICVCEY | Deltaprotesin |

## Supplementary Table S6. Class V peptides.

| ID | Signal Peptide | Mature Sequence | Name | NCBI Taxonomy ID |
|---|---|---|---|---|
| **WP_152921545.1** | *MKKVSGLCFLLLVLFVAQEIVVQTEA* | RTCENLADKYKGPCFTDGSCDDHCKNKAHLISGRCIKIRCWCTRNC | Colisin-1 | txid562 |
| **WP_152932525.1** | *MKKVSGLCFLLLVLFVAQEVVVQTEA* | RTCENLADKYRGPCFTDGSCDDHCKNKAHLISGRCIKIRCWCTRNC | Colisin-2 | txid562 |
| **WP_152922005.1** | *MKKVSGLCFLLLVLFVAQEIVVQTEA* | RTCENLADKYKGPCFGGCDSHCRNKEHLLSGRCRDDFRCWCTRNC | Colisin-3 | txid562 |
| **WP_152928696.1** | *MKKVSGLCFLLLVLFVAQEIVVQTEA* | RTCENLADKYRGPCFGGCDSHCRNKEHLLSGRCRDDFRCWCTRNC | Colisin-4 | txid562 |
| **WP_152930364.1** | *MKKVSGLCFLLLVLFVAQEVVVQTEA* | RTCENLADKYRGPCFTNGSCDDHCKNKEHLLSGRCRDDFRCWCTRNC | Colisin-5 | txid562 |
| **WP_152933380.1** | *MKKVSGLCFLLLVLFVAQE* | IVVQTEARTCENLADTYKGPCFTTGSCDDHCKNKEHLLSGRCRDDFRCWCTRNC | Colisin-6 | txid562 |
| **WP_083513115.1** | *MAKFASIIAPLFAALVLFAAFEAPTMVEA* | QKLCERPSGTWSGVCGNNNACKNQCINLEKARHGSCNYVFPAHKCICYFPC | Barkerisin-1 | txid33917, txid3711, txid3708 |
| **WP_082181585.1** | *MAGNGVGTRLSALFLLVLLVITIGMMDVQVAEG* | RMCKTPSGKFKGYCVSSTNCKNVCRTEGFPTGSCDFHITSRKCYCYKPCP | Baumaniisin-1 | txid470 |
| **WP_082181597.1** | *MEGKGVGTRLSALFLVVLLVISIGMMEVQVAEG* | RMCKTPSGKFKGYCVSSTNCKNVCRTEGFPTGSCDFHVASRKCYCYKPCP | Baumaniisin-2 | txid470 |
| **WP_151421658.1** | *MPLVSTVFLVLMLLMATEMGGRLAEG* | AKCDQPSGNFKGPCSTLTPDCSNTCHGEGFTLGGHCTNFRCVCTKPCPDN | Baumaniisin-3 | txid470 |
| **WP_147407704.1** | *SISCLLIQKQKMKGSQRLFSAFLLVILLFMATDMGPMVTEA* | RTCESQSHRFKGTCVRQSNCAAVCQTEGFHGGNCRGFRRRCFCTKHC | Pylorisin-1 | txid210 |
| **RKV06550.1** | *MKGSQRLFSAFLLVILLFMATDMGPMVTEA* | RTCESQSHRFKGTCVRQSNCAAVCQTEGFHGGNCRGFRRRCFCTKHC | Pylorisin-2 | txid210 |
| **WP_109042593.1** | *MKQSMRPFATIFLVLLLVLAAEVGPRVAEA* | RTCGTASQRFRGLCLRKRNCEAVCNTEGYPGGSCQGFRRRCICNRPCE | Pneumonisin-1 | txid573 |
| **WP_149346031.1** | *MARSKSLVPTIFFFLLLLVATEMVAEA* | RTCESKSHRFKGPCVRDSNCASVCETEHFSGGNCRGFRRRCFCTKHCP | Hormaechesin-1 | txid158836 |
| **KTD21282.1** | *MNLKKTLSALFLMACAMNSYAHG* | DQDTYTIPIGTKCISKDTRHCTDACKSNGFEKGICKFPNPNIDMGRCYCRYY | Londiniensisin-1 | txid45068 |

**Supplementary Table S7. Potential *Actinomyces* defensin-like sequences discarded in signal peptide prediction step.**

| NCBI ID | Species | Sequence |
|---|---|---|
| >WP_009234272.1 | *Actinomyces, Actinomyces sp. oral taxon 849 str. F0330, Actinomyces johnsonii F0542* | MSQFIRRTSTLTDISFSDALHSESHMPLEGAEGPCPHNETKCGEVCRGMGYTGGYCHSWFNLICKCY |
| >WP_119836391.1 | *Actinomyces sp. 2119* | MPHFIRRTTTLAGADFTQALRSETHAPTEGAEPFGCPALEFVCNRHCRSIARNYYKGKCVGMFKQTCKCFSY |
| >WP_119836392.1 | *Actinomyces sp. 2119* | MSQFIRRTTALAGADFTQALRSETHAPTEGGEPFGCPFNSFTCHRHCKSIPGYRGGYCKGRLNQTCKCYR |
| >WP_119836390.1 | *Actinomyces sp. 2119* | MHPLIRRTTALAGADFAQALRSETHAPVEGSESFPCLGHPARCFAHCRKAGFRGGYCVPIRRCVCY |
| >WP_120203678.1 | *Actinomyces sp. 2129* | MSQFIRRTTTLAGADFTQALRSETHAPTEGAEGPCPLNEKKCSQICRAKGYKGGYCGSFANLVCKCY |
| >WP_127842650.1 | *unclassified Actinomyces* | MPPFIRRTTALAGADFTQALRSETHAPVEGSESFPCLGHPARCVAHCRKVGFRGGYCVPIRRCVCY |
| >WP_127842649.1 | *unclassified Actinomyces* | MSPFIRRTTALAGADFTQALRSETHAPTEGAEDFGCGPFVGNGKCRRHCRNTGRRDGYCMGMFKQTCKCHG |
| >WP_127842648.1 | *Actinomyces sp. 299* | MSQFIRRTTALAGADFSQALRSETHAPTEGAEGPCPHNEGKCNRICKAKGYHGGYCGSFANLVCKCYG |
| >WP_139738766.1 | *Actinomyces sp. 340* | MSQFIRRTTTLAGADFTQALRSETHVPAEGAEPFGCPFNQYECHRHCRSAGFRGGYCKGMFKQTCKCYG |
| >WP_141425203.1 | *Actinomyces johnsonii* | MDKFTRRTAPLSDADSAQAISSETQAPIEGAEGFGCPNEYSCNAHCRGNGFRGGYCDSWFRLRCHCY |
| >WP_141407487.1 | *Actinomyces oris* | MSKIRKHPPVRQQFPHPTHRTRMKKEIYMELFSRRCKSLSDSRFSDAMNAETRNPLETRNCFACPFNEHQCHNHCLSTGYRGGFCGGFAAATCRCY |

| | | |
|---|---|---|
| >WP_141407488.1 | *Actinomyces oris* | MDKFTRRTTTLSDTDFNQAVTSETQAPIEGAEGFSCPGAEYACNNRCRSIGYRGGYCGSLFNLHCHCY |
| >WP_141407489.1 | *Actinomyces oris* | MDKFTRRTATLSDTDFNQAVTSETQAPIEGTEDLSCPWAERVCDTHCRSIGYRGGYCAGPIKLVCHCY |
| >WP_141407492.1 | *Actinomyces oris* | ALHSETHPPTEGAEYNCPTDESPCDRHCRHSGYRGGYCGGALKTSCRCY |
| >WP_141759000.1 | *Actinomyces sp. HMSC075C01* | MEGAEGIGCPGAEYGCNKRCRSIGYRGGYCGSLFNLRCHCY |
| >WP_143226823.1 | *Actinomyces naeslundii* | MSRFIRRSRSLANINLSSTLRNEMQPPLEANGLGCPNEWECDSRCRVDGARGGYCDAWTLWTTCKCY |
| >WP_147680917.1 | *Actinomyces ruminicola* | MKKFIRRSSSLAAASFEQAFQSETQVPLEGAEGFGCNLITSNPYQCSNHCKSVGYRGGYCKLRTVCTCY |
| >KAA8739320.1 | *Actinomyces johnsonii* | GAEGFGCPNEYSCNAHCRGNGFRGGYCDSWFRLRCHCY |
| >ERH18088.1 | *Actinomyces johnsonii F0510* | MKAGNRHSGGCAVEAECRSCCPDRLSSPIPGCQASCPLRCRCRLPAH |
| >WP_021605874.1 | *Actinomyces johnsonii, Actinomyces johnsonii F0510* | MKKEILMELFSRRRKSLSDSRFNDTMNAETRIPLETSDCFACPFNEQQCHNHCLSTGYRGGFCGGFAAATCRCY |
| >WP_053587718.1 | *Actinomyces sp. oral taxon 414* | MKFIRRSNNLTDVSFTQALHSETRAPLEGAEGYGCSPLSSDYQCTEHCRYIGYRGGYCAWGIVCTCY |
| >WP_070658096.1 | *Actinomyces, Actinomyces sp. HMSC075C01,* | MDKFTRRTTTLSDSDFSQAVSSETQAPIEGAEGFSCPGAEYACNNRCRSIGYRGGYCGSWLNLQCRCY |

| | | |
|---|---|---|
| >WP_070658097.1 | *Actinomyces oris Actinomyces, Actinomyces sp. HMSC075C01, Actinomyces oris* | MDKFTRRTANLVDADKALNAETHAPIEGAEGFGCPWNAYECDRHCMSKGYTGGNCRGKIRQTCHCY |
| >WP_073451524.1 | *Actinomyces, Actinomyces denticolens, Actinomyces sp. Chiba101* | MKQFARRTATLADATFTQALDSETKPPTEGAEFSCPMTDYPCIMHCKAIGYRGGYCGGFLNLSCRCH |
| >WP_073451525.1 | *Actinomyces, Actinomyces denticolens, Actinomyces sp. Chiba101* | MDKFTRRTADLAANELGDDINAETRTPLEDAEGFGCPFNAYQCHSHCLSIGRRGGYCRGLVRQTCVCYR |
| >CED90094.1 | *Actinomyces succiniciruminis* | MKNFIRRSSSLDAVSFEQALRSETHAPLEGAEGFGCPFSERSCDTHCMTKGYRGGYCKGAVRQTCVCYK |
| >CED90093.1 | *Actinomyces succiniciruminis* | MKKFIRRSSSLDAVSFEQALKSETHVPLEGAEGFGCPFRPGDCYKYCRSKGFRVGVCDSLANMRCHCYY |
| >WP_075250110.1 | *Actinomyces oris* | MDKFTRRAAPLSDASFNQAISSETQAPIEGAEYGCPGAEYGCNNRCRSIGYRGGYCGSLFNLRCLCY |
| >WP_075250109.1 | *Actinomyces oris* | MDKFTRRTAPLSDADSKHAISSETQAPIEGAEGFGCPNEYRCNAHCQSVGYQGGYCDFWTARRRCTCY |
| >WP_075250111.1 | *Actinomyces oris* | MPRFVRRSTALADVTFAQALHSETHAPTEGAEYNCPTDESPCDRHCRYSGYRGGYCGGALKTSCHCY |
| >WP_075250112.1 | *Actinomyces oris* | MDKFTRRTASLSDSDFKQAISSETHAPIEGAEGFGCPDESRCNAHCQNNGFDRGRCDSIFALRCHCSYYR |

| | | |
|---|---|---|
| >OLL13937.1 | *Actinomyces oris* | MELFSRRCKSLSDSRFSDAMNAETRNPLETSDCFACPFNEHQCHNHCLSTGYRGGFCGGFAAATCRCY |
| >WP_075371451.1 | *Actinomyces oris* | MDKFTRRTTTLSDSDFSQAVSSETQAPIEGAEGIGCPGAEYGCNKRCRSIGYRGGYCGSLFNLRCHCY |
| >OLO50376.1 | *Actinomyces oris* | MELFSRRCTSLSDSRFSDTMNAETRNPLETSDCFACPFNEHQCHNHCLSTGYRGGFCGGFAAATCRCH |
| >WP_075371330.1 | *Actinomyces, Actinomyces oris* | MPHFVRRSTALADVTFEQALHSETHTPTEGAEYKCPTDESPCDRHCRYSGYRGGYCGGILKTSCRCY |
| >OLO51711.1 | *Actinomyces oris* | MDMFSRRRTSLSDNRFRGTMNAETRNPLETSNCFACPFNEHQCHNHCLSKGYRGGFCGGFAAATCRCH |
| >WP_075372939.1 | *Actinomyces oris* | MDKFTRRTAPLSDADFKQAVSSETQAPIEGAEGFGCPNDEYTCNAHCQSVGYRGGYCDFWTAWRRCTCY |
| >WP_075372941.1 | *Actinomyces oris* | MPRFVRRSTALADVTFAQALHSETHPPTEGAEYNCPTDESPCDRHCRYSGYRGGYCGGALKTSCRCY |
| >WP_075372942.1 | *Actinomyces oris* | MDKFTRRTAPLSDADFKQAVSSETQAPIEGAEGFGCPNDEYTCNAHCQSVGYRGGYCDFWTGWRRCTCY |
| >WP_075372938.1 | *Actinomyces oris* | MPQFVRRSTALADVTFEQALHSETHAPTEGAEYNCPTDESPCDRHCRYSGYRGGYCGGALKTSCRCY |
| >WP_075372937.1 | *Actinomyces oris* | MDKFTRRTAPLSDADFKQAVSSETQAPIESAEGHGCPADEYRCYRDCRAMGYRGGYCDSRTLWLRCTCY |
| >WP_075372940.1 | *Actinomyces oris* | MDKFTRRTAPLSAADFKQAISSETQAPIEGTEGFSCPGAEYACNAHCRSIGYRGGYCGSWLNLRCRCY |
| >OLO60437.1 | *Actinomyces oris* | MELFSRRCKSLSDSRFSDAMNAETRNPLETSNCFACPFNEHQCHNHCLSTGYRGGFCGGFAAATCRCY |
| >OMG10321.1 | *Actinomyces naeslundii* | MSQFIRRSSKLTNVTFNQSLLSETCPPLEGGEGFGCPGQEYWCDGHCKANGFQYGKCDSLFWHRCHCFE |
| >WP_076067805.1 | *Actinomyces naeslundii* | MNRFVRRSRSLADVTFASVLRSETHAPLEGAEGHGCRPFGSDFECDEWCTYKGYRGGYCSWGVVCTCYGG |

| | | |
|---|---|---|
| >WP_081376788.1 | *Actinomyces oris* | MPKIRKHPPVRQQLPHPTHRTRMKKEIYMELFSRRCKSLSDSRFSDAMNAETRNPLETSDCFACPFNEHQCHNHCLSTGYRGGFCGGFAAATCRCY |
| >WP_081381864.1 | *Actinomyces oris* | MSKIRKHPPVQQQFPHPTHRTRMKKEIYMELFSRRCKSLSDSRFSDAMNAETRNPLETSNCFACPFNEHQCHNHCLSTGYRGGFCGGFAAATCRCY |
| >WP_081384611.1 | *Actinomyces oris* | MKKENYMDMFSRRRTSLSDNRFRGTMNAETRNPLETSNCFACPFNEHQCHNHCLSKGYRGGFCGGFAAATCRCH |
| >WP_081384623.1 | *Actinomyces* | MPKIRKHSPVRKQLPHPTHRTHMKKENYMELFSRRCTSLSDSRFSDTMNAETRNPLETSDCFACPFNEHQCHNHCLSTGYRGGFCGGFAAATCRCH |
| >WP_082463042.1 | *Actinomyces johnsonii* | MPRFVRRSTALADVTFEQALHSETHAPTEGAEYNCPTDESPCDRHCRYSGYRGGYCGGILKTSCRCY |
| >WP_086615404.1 | *Actinomyces sp. CtC 72* | MRKFIRRSSSLAAASFEQALRSETQAPLEGAEGFGCPNEYKCNRHCKSVNYRGGYCDFWTARLRCTCY |
| >WP_087943935.1 | *Actinomyces sp. oral taxon 171, Actinomyces sp. oral taxon 171 str. F0337* | MDMFSRRRKSLNDSRFNDAMNAETRSPLETSDCFACPFNEHQCHNHCLSKGYRGGFCGGFAAATCRCH |
| >WP_087943934.1 | *Actinomyces sp. oral taxon 171, Actinomyces sp. oral taxon 171 str. F0337* | MPHFVRRSTALADVTFEQALHSETHTPTEGAEYNCPTDESPCDRHCRYSGYRGGYCGGILKTSCRCY |
| >WP_009393171.1 | *Actinomyces sp. oral taxon 171, Actinomyces sp. oral taxon 171 str. F0337* | MDKFTRRTTTLSDSDFSQAVSSETQAPIEGTEDLSCPWAPSVCNRHCLSHGYRGGYCAGPIKLVCHCY |

| | | |
|---|---|---|
| >WP_009393172.1 | *Actinomyces sp. oral taxon 171, Actinomyces sp. oral taxon 171 str. F0337* | MDKFTRRTANLVDADKALNAETHAPIEGAEGFGCPWNAYECDRHCVSKGYTGGNCRGKIRQTCHCY |

**Supplementary Table S8. Summary of the minimal inhibitory concentration (MIC) results of xanthusin-1.**

| Microorganism | MIC |
|---|---|
| *Escherichia coli* ATCC 25922 | >128 μg $mL^{-1}$ |
| *Klebisiella pneumoniae* ATCC 13883 | >128 μg $mL^{-1}$ |
| *Staphylococcus aureus* ATCC 25923 | >128 μg $mL^{-1}$ |
| *Candida albicans* ATCC 10231 | >128 μg $mL^{-1}$ |
| *Criptococcus neoformans* H99 | >128 μg $mL^{-1}$ |

**Supplementary Table S9. Summary of transcriptomic data analyzed.**

| ID* | Submission Year | Conditions | Experimental n | Location |
|---|---|---|---|---|
| SRP156251 | 2018 | Presence and abscence of chloramphenicol | 2 | Shandong University |
| SRP024249 | 2013 | Casitone-based growth media | 3 | UCDavis |
| SRP180970 | 2019 | Low nutriente availability | 2 | Universidad de Granada |

*ID from SRA database.
